# Differential synaptic depression mediates the therapeutic effect of deep brain stimulation

**DOI:** 10.1038/s41593-025-02088-w

**Published:** 2025-10-16

**Authors:** Jicheng Li, Jingheng Zhou, Bo He, Amy B. Papaneri, Nicholas P. Kobzar, Alan C. Yeh, Ying Zhang, Guohong Cui

**Affiliations:** 1 In Vivo Neurobiology Group, Neurobiology Laboratory, National Institute of Environmental Health Sciences, National Institutes of Health, Research Triangle Park, Durham, NC USA; 2 Viral Vector Core, National Institute of Environmental Health Sciences, National Institutes of Health, Research Triangle Park, Durham, NC USA; 3 Department of Neurology and Neuroscience Center, The First Hospital of Jilin University, Changchun, China

**Keywords:** Parkinson's disease, Parkinson's disease

## Abstract

Deep brain stimulation (DBS) effectively treats drug-resistant neurological and psychiatric disorders, yet its mechanisms remain unclear. Here we show that high-frequency DBS of the subthalamic nucleus (STN), a common target for Parkinson’s disease (PD), activates afferent axons while inhibiting STN neurons. These contrasting presynaptic and postsynaptic effects arise from a decrease in local neurotransmitter release with a larger decrease in glutamate than GABA, shifting the excitation/inhibition balance toward inhibition. Chemogenetic inhibition, but not excitation, of STN neurons mimics the therapeutic effects of DBS in 6-OHDA-lesioned PD mice. Acute and chronic bilateral chemogenetic STN inhibition restores motor function in a progressive PD mouse model. These findings suggest that inhibition of STN, caused by differential depression of glutamatergic and GABAergic synapses, is a key mechanism of therapeutic DBS. ‘Chemogenetic DBS’, direct chemogenetic inhibition of postsynaptic neurons, may offer a less invasive and more affordable alternative to electrical DBS for PD and other neurological disorders.

## Abstract (web summary)

The authors show that deep brain stimulation (DBS) inhibits local neural activity via differential suppression of glutamate and GABA release, and they developed a chemogenetic therapy as a less invasive, cost-effective alternative to electrical DBS.

## Main

DBS is a surgical intervention that delivers high-frequency electrical pulses through electrodes implanted in targeted brain structures to mitigate the symptoms in refractory neurological and psychiatric conditions^1^. DBS in the STN or the globus pallidus internal segment (GPi) has become a standard treatment for medication-resistant, advanced PD^2–4^. Despite its effectiveness in alleviating symptoms of PD and other disorders, DBS is an invasive and costly procedure with inherent risks, including hemorrhage and infection, associated with the lifelong placement of electrodes in the brain and the pulse generator and wires under the skin. Understanding the mechanisms of DBS is crucial for developing more effective treatments with higher efficacy and a lower risk of complications. However, a unifying hypothesis explaining the mechanisms behind its beneficial outcomes is still lacking^1,5–8^. In the first successful cases of treating patients with PD with STN DBS, inhibition was proposed as the primary mechanism of action^2,9^. This hypothesis arose from observations that the STN exhibits hyperactivity in primate models of PD^10^ and that lesioning, the ultimate form of inhibition, of the STN alleviates PD symptoms^11^. This ‘reversible lesion’ hypothesis of STN DBS was further supported by findings indicating that patients with PD exhibited increased activity in STN neurons^12^ and that both lesioning and DBS produced similar effects on the output nuclei of the basal ganglia^13,14^. However, this hypothesis was later challenged by conflicting reports on the local effects of STN DBS^15,16^, the fact that DBS also modulates network activity by activating afferent and passing fibers^7,17^ and the mixed results from optogenetic and chemogenetic studies^18–21^. The current state of DBS research suggests a multifactorial mechanism, with a particular emphasis on modulation of the complex cortical-basal ganglia-thalamic network^1,6,7^. This study aims (1) to uncover the neuronal mechanisms of DBS using spectrally resolved fiber photometry^22^ paired with genetically encoded fluorescent sensors expressed strategically to target presynaptic and postsynaptic components and neurotransmitters in the stimulating site and (2) to develop a less invasive and more affordable alternative treatment based on the mechanisms of electrical DBS.

## Results

### Effect of DBS on STN neuronal activity

**Extended Data Fig. 1:**
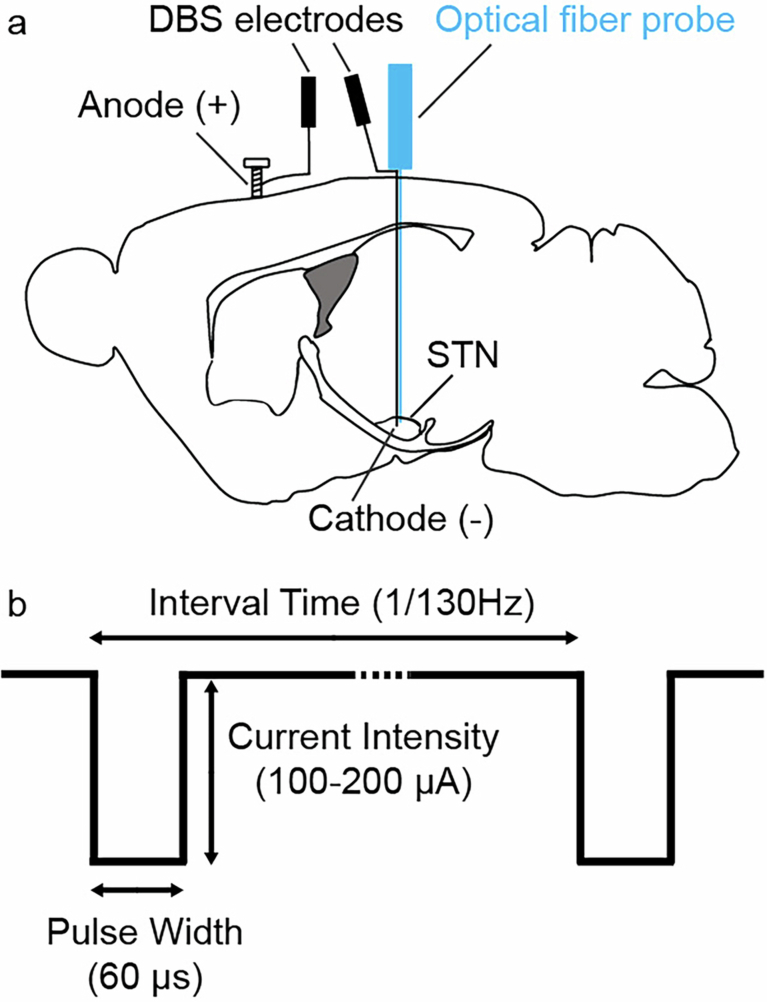
Illustration of monopolar monophasic cathodic STN DBS. **a**, Schematic illustration of the DBS electrode configuration. An insulated tungsten wire with exposed contacts at both ends is implanted in the brain, targeting the STN, and serves as the cathode during stimulation. An uncoated stainless-steel wire is wrapped around an anchoring screw on the skull and serves as the anode. **b**, Diagram showing the waveform and parameters of the DBS stimulation pulses.

We selected the STN as the target for DBS in this study because it is considered a preferred site for most patients with PD owing to its superior effects on motor improvement and medication reduction^1^. We used the most common stimulating mode^23^ (monopolar, monophasic, cathodic stimulation) and parameters (130 Hz, 60-μs pulse width; Extended Data Fig. 1) that are proven effective in alleviating motor symptoms in both human patients and rodent studies^2,24^.

**Fig. 1:**
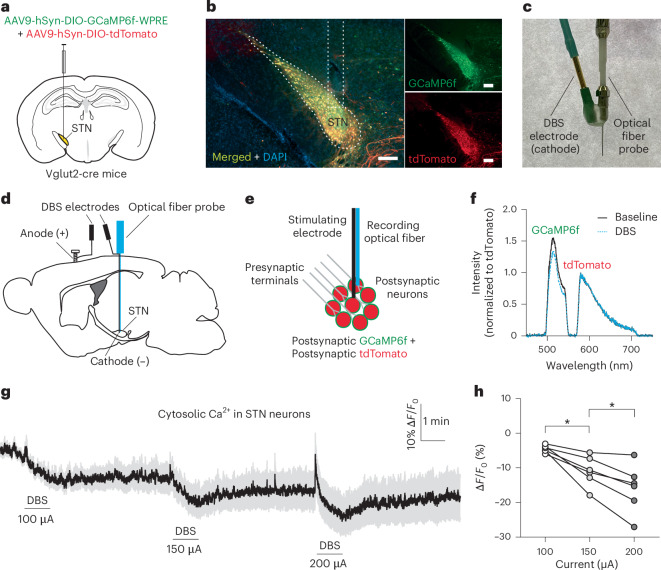
High-frequency STN DBS causes sustained inhibition in STN neurons after initial excitation in normal mice. **a**, Schematic illustration showing microinjection of AAVs to express GCaMP6f and tdTomato in STN neurons in Vglut2-cre mice. **b**, Representative images to show the expression of GCaMP6f and tdTomato in the STN. Scale bar, 200 μm. **c**, A picture of a lab-made hybrid probe consisting of an optical fiber for photometry recordings and an electrode for monopolar DBS stimulation. The two indicator lines point to the gold connector pin soldered to the insulated tungsten wire, which serves as the cathode during monopolar DBS stimulation, and to the ceramic ferrule of the optical fiber probe, respectively. The electrode and fiber probe are secured together using Metabond dental adhesive. A schematic illustration of this electrode–optical fiber hybrid probe is shown in **d**. **d**, Schematic illustration of fiber photometry recordings during monopolar DBS stimulation in the STN in vivo. The hybrid probe shown in **c** is implanted to target the STN. A stainless steel wire secured to the anchoring screw on the skull serves as the anode during DBS. **e**, Illustration of fiber photometry recordings of postsynaptic neural activity in the STN during STN DBS using GCaMP6f and tdTomato co-expressed in STN neurons. tdTomato serves as a control fluorescent protein for ratiometric measurement of cytosolic Ca^2+^. **f**, Representative emission spectra of GCaMP6f and tdTomato recorded from STN neurons before and during DBS stimulation. **g**, Time-lapsed ratio of GCaMP6f/tdTomato fluorescence (normalized to the baseline before the first DBS stimulation) to show the DBS-induced responses in STN neurons at different stimulation intensities. **h**, Summary of DBS-induced decreases in the cytosolic Ca^2+^ in STN neurons at different stimulation intensities. *P < 0.05 (100 µA versus 150 µA, *P* = 0.0178; 150 µA versus 200 µA, *P* = 0.0153), *n* = 6 mice, including both males and females, repeated one-way ANOVA followed by Dunnett’s multiple comparisons test. Source data

**Extended Data Fig. 2:**
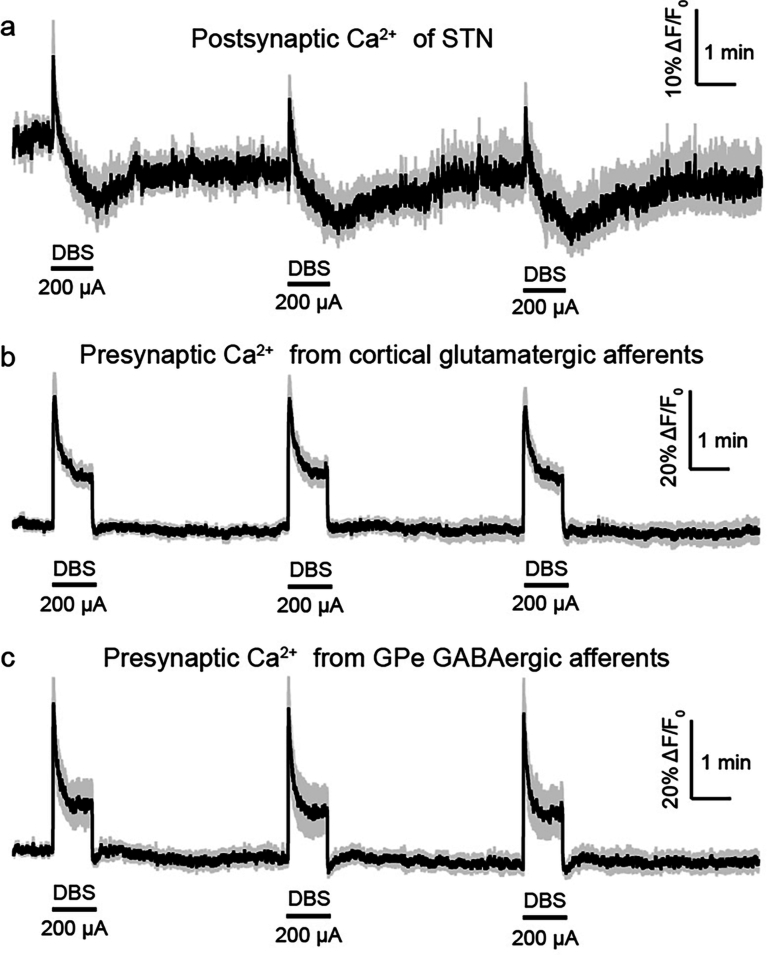
Effects of High-frequency STN DBS on intracellular Ca^2+^ in STN neurons and afferent presynaptic terminals in the STN of normal mice. **a**, Time-lapsed ratio of GCaMP6f/tdTomato fluorescence (normalized to the baseline before the first DBS stimulation) to show the DBS-induced responses in STN neurons; stimulation repeated 3 times with 200 µA current. **b**, **c**, Time-lapsed ratio of GCaMP8f/tdTomato fluorescence (normalized to the baseline before the first DBS stimulation) to show the DBS-induced changes in presynaptic Ca^2+^ levels in glutamatergic (**b**) and GABAergic (**c**) afferent terminals in the STN; stimulation repeated 3 times with 200 µA current. Source data

**Extended Data Fig. 3:**
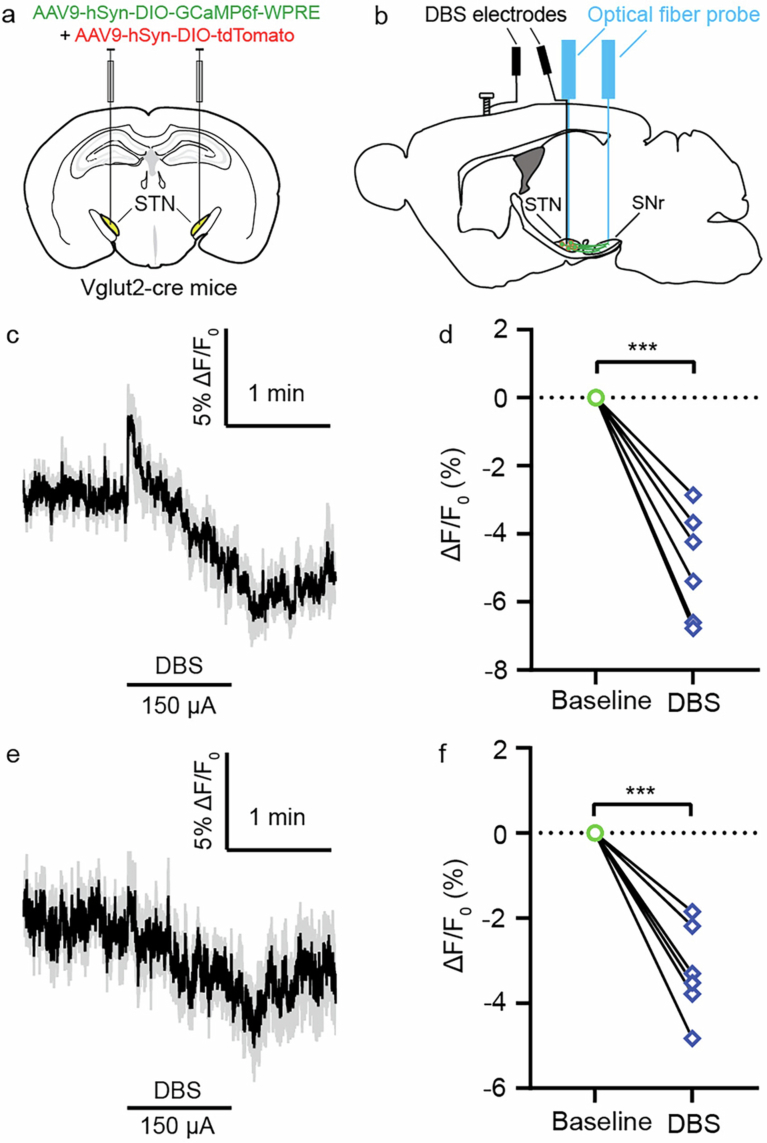
Simultaneous paired fiber photometry recordings of intracellular Ca^2+^ from the somata of STN neurons and their axonal terminals in the SNr of normal mice during high-frequency STN DBS. **a**, Schematic illustration showing bilateral microinjections of AAVs to express GCaMP6f and tdTomato in STN neurons. **b**, Schematic illustration of simultaneous paired fiber photometry recordings in the STN and in the SNr during STN DBS. **c**, **e**, Time-lapsed normalized ratio of GCaMP6f/tdTomato fluorescence (plotted as mean ± SEM) to show the STN DBS-induced intracellular Ca^2+^ in the somata of STN neurons (**c**) and their axon terminals in the SNr (**e**). **d**, **f**, Summary of DBS-induced changes in the fluorescence ratio of GCaMP6f/tdTomato (normalized to the baseline before the stimulation) in the somata of STN neurons (**d**) and their axon terminals in the SNr (**f**). *** p < 0.001 (For **d**, p = 0.0006; for **f**, p = 0.0008), paired two tailed t test, n = 6 from 5 mice (one mouse received bilateral implantation with one pair of probes in each hemisphere), including both males and females. Source data

To evaluate the postsynaptic effect of high-frequency DBS on STN neurons, we co-expressed GCaMP6f and tdTomato in STN neurons by injecting AAV9-hSyn-DIO-GCaMP6f-WPRE and AAV9-hSyn-DIO-tdTomato into the STN of Vglut2-cre mice (Fig. 1a,b) and implanted a hybrid probe consisting of a stimulating electrode and an optical fiber probe into the STN (Fig. 1c,d). In this preparation, the green GCaMP6f fluorescence collected by the optical fiber, which is parallel to but with its tip placed 0.1 mm to 0.2 mm above the tip of the stimulating electrode, reports the neural activity in STN neurons while the red tdTomato fluorescence serves as the control (Fig. 1e). Using spectrally resolved fiber photometry^22^, we were able to obtain a series of time-lapsed fluorescence emission spectra of GCaMP6f and tdTomato before and during DBS stimulation in freely moving mice (Fig. 1f). We found that, during DBS stimulation, the ratio of GCaMP6f/tdTomato fluorescence (F_GCaMP6f/tdTomato_) showed sustained decrease after a brief initial rise (Fig. 1g and Extended Data Fig. 2a). The magnitude of decrease, measured by the percent change of F_GCaMP6f/tdTomato_, was correlated with the intensity of the stimulation (100 µA: −4.523 ± 0.4202; 150 µA: −10.94 ± 1.771; 200 µA: −15.87 ± 2.846; Fig. 1h). These results suggest that STN neurons are persistently inhibited after a brief activation during prolonged high-frequency DBS. To further investigate whether DBS-induced inhibition in the somata of STN neurons extends to their axonal terminals and to test the soma−axon ‘decoupling’ hypothesis^25^, we conducted simultaneous paired fiber photometry recordings from both the somata of STN neurons and their axonal terminals in the substantia nigra pars reticulata (SNr) (Extended Data Fig. 3a,b). Our findings revealed that the activity of the axonal terminals was also inhibited during STN DBS (Extended Data Fig. 3c–f).

### Effect of DBS on afferent terminal activity

Next, to investigate how DBS affects the activity in afferent axon fiber terminals, we expressed GCaMP8f in the neurons that project to the STN and tdTomato in the STN neurons as a fluorescence control and a guide for precisely targeting the STN during fiber implantation surgeries. Because the STN receives both glutamatergic inputs from multiple cortical areas and GABAergic inputs from the globus pallidus external segment (GPe)^26^, we examined the glutamatergic axonal terminals and GABAergic axonal terminals separately in two sets of experiments.

**Fig. 2:**
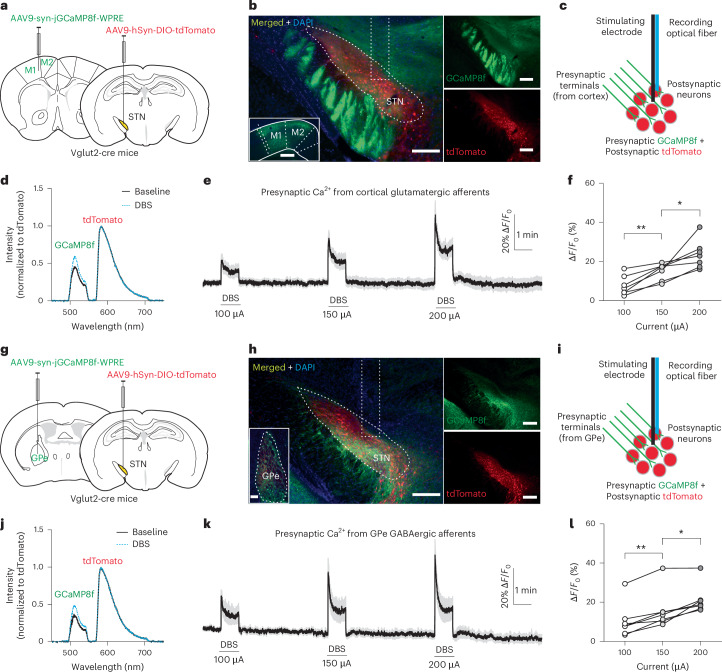
High-frequency STN DBS causes sustained excitation in glutamatergic and GABAergic afferent terminals in the STN of normal mice. **a**,**g**, Schematic illustrations of the strategies to express GCaMP8f in the glutamatergic (**a**) and GABAergic (**g**) afferent terminals in the STN and tdTomato in STN neurons. **b**,**h**, Immunofluorescence images to show the expression of GCaMP8f in M1 and M2 cortical (small insert in **b**) and GPe (small insert in **h**) neurons and in their axonal terminals projecting to the STN and the expression of tdTomato in STN neurons. Scale bars, 200 μm, except the small insert in **b**, which is 500 μm. **c**,**i**, Illustrations of fiber photometry recordings of presynaptic Ca^2+^ levels in the STN during STN DBS using GCaMP8f expressed in glutamatergic (**c**) and GABAergic (**i**) afferent terminals and tdTomato expressed in STN neurons. tdTomato serves as a control fluorescent protein and a marker for STN to guide the probe implantation. **d**,**j**, Representative emission spectra of GCaMP8f expressed by glutamatergic (**d**) and GABAergic (**j**) axonal terminals and tdTomato expressed by STN neurons before and during DBS stimulation. **e**,**k**, Time-lapsed ratio of GCaMP8f/tdTomato fluorescence (normalized to the baseline before the first DBS stimulation) to show the DBS-induced changes in presynaptic Ca^2+^ levels in glutamatergic (**e**) and GABAergic (**k**) afferent terminals in the STN at different stimulation intensities. **f**,**l**, Summary of DBS-induced increases in presynaptic Ca^2+^ levels in glutamatergic (**f**) and GABAergic (**l**) afferent terminals in the STN at different stimulation intensities. *P < 0.05, **P < 0.01 (for **f**: 100 µA versus 150 µA, *P* = 0.0025; 150 µA versus 200 µA, *P* = 0.0274; for **l**: 100 µA versus 150 µA, *P* = 0.0019; 150 µA versus 200 µA, *P* = 0.0164), *n*= 7 mice for each group, including both males and females, repeated one-way ANOVA followed by Dunnett’s multiple comparisons test. Source data

To express GCaMP8f in glutamatergic afferents into the STN and tdTomato in STN neurons, we injected AAV9-syn-jGCaMP8f-WPRE into the M1 motor cortex and AAV9-hSyn-DIO-tdTomato into the STN of Vglut2-cre mice and implanted the electrode−optical fiber hybrid probe into the STN (Fig. 2a−c). In contrast to the postsynaptic inhibitory effect on local STN neurons, DBS produced sustained activation of presynaptic terminals reported by an elevated F_GCaMP8f/tdTomato_, and the magnitude of activation, measured by the percent change of F_GCaMP8f/tdTomato_, correlated with the intensity of the stimulation (100 µA: 7.687 ± 1.898; 150 µA: 15.09 ± 1.607; 200 µA: 23.52 ± 2.764; Fig. 2d−f and Extended Data Fig. 2b).

To express GCaMP8f in GABAergic afferents into the STN and tdTomato in STN neurons, we injected AAV9-syn-jGCaMP8f-WPRE into the GPe and AAV9-hSyn-DIO-tdTomato into the STN of Vglut2-cre mice and implanted the electrode−optical fiber hybrid probe into the STN (Fig. 2g−i). Similar to the effects on glutamatergic afferents, DBS produced sustained activation of GABAergic presynaptic terminals, reported by an elevated F_GCaMP8f/tdTomato_, and the magnitude of activation, measured by the percent change of F_GCaMP8f/tdTomato_, correlated with the intensity of the stimulation (100 µA: 10.38 ± 3.349; 150 µA: 15.86 ± 3.691; 200 µA: 21.28 ± 2.785; Fig. 2j−l and Extended Data Fig. 2).

**Extended Data Fig. 4:**
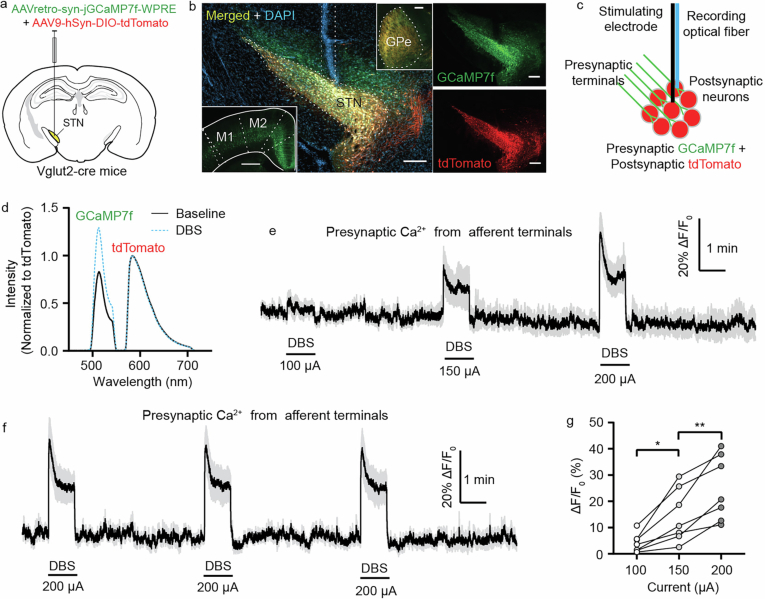
High-frequency STN DBS excites the presynaptic afferent terminals in the STN of normal mice. **a**, Schematic illustration of the strategy to express GCaMP7f in the afferent terminals in the STN and tdTomato in STN neurons of Vglut2-cre mice. **b**, Immunofluorescence images to show the expression of GCaMP7f in the cortex and GPe, and GCaMP7f and tdTomato in the STN. Scale bars: 200 μm, except in the small insert of Cortex (M1/M2), which is 500 μm. **c**, Illustrations of fiber photometry recordings of presynaptic Ca^2+^ levels in the STN during STN DBS using GCaMP7f expressed in afferent terminals and tdTomato expressed in STN neurons. tdTomato serves as a control fluorescent protein and a marker for STN to guide the probe implantation. **d**, Representative emission spectra of GCaMP7f expressed by presynaptic axonal terminals and tdTomato expressed by STN neurons before and during DBS stimulation. **e**, **f**, Time-lapsed ratio of GCaMP7f/tdTomato fluorescence (normalized to the baseline before the first DBS stimulation) to show the DBS-induced changes in presynaptic Ca^2+^ levels in afferent terminals in the STN at different stimulation intensities (**e**) and at the same intensity (**f**). **g**, Summary of DBS-induced increases in presynaptic Ca^2+^ levels in afferent terminals in the STN at different stimulation intensities. * p < 0.05, ** p < 0.01 (100 µA vs. 150 µA, p = 0.0198; 150 µA vs. 200 µA, p = 0.0081), Repeated one-way ANOVA followed by Dunnett’s multiple comparisons test. All data are plotted as mean ± SEM. n = 7 mice for each group, including both males and females. Source data

**Extended Data Fig. 5:**
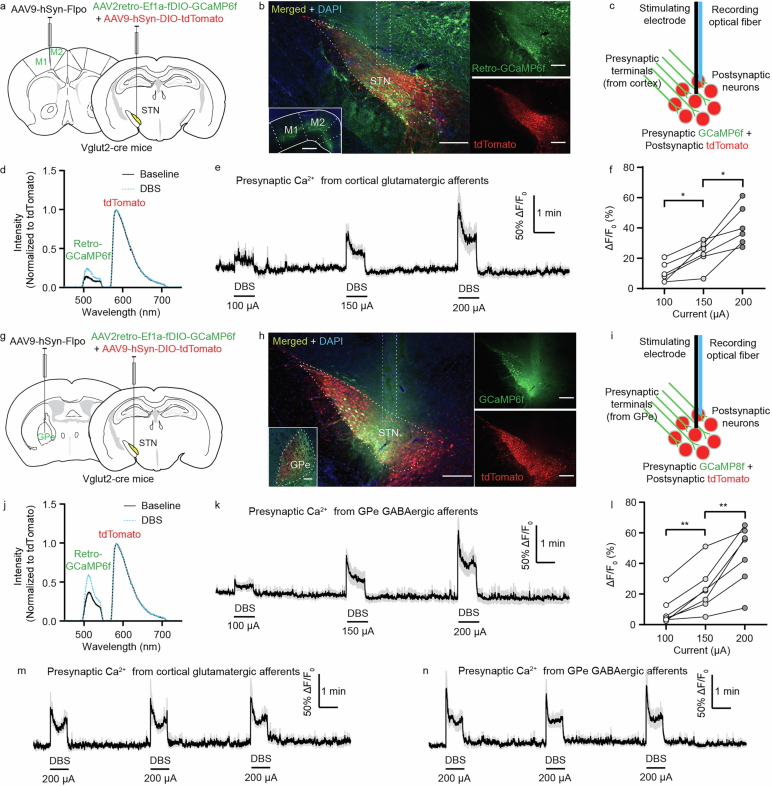
High-frequency STN DBS excites glutamatergic and GABAergic afferent terminals in the STN of normal mice. **a**, **g**, Schematic illustrations of the strategies to express GCaMP6f in the glutamatergic (**a**) and GABAergic (**g**) afferent terminals in the STN and tdTomato in STN neurons of Vglut2-cre mice. **b**, **h**, Immunofluorescence images to show the expression of GCaMP6f in M1 and M2 cortical (small insert in **b**) and GPe (small insert in **h**) neurons and in their axonal terminals projecting to the STN, and the expression of tdTomato in STN neurons. Scale bars: 200 μm except in the small insert in **b**, which is 500 μm. **c**, **i**, Illustrations of fiber photometry recordings of presynaptic Ca^2+^ levels in the STN during STN DBS with GCaMP6f expressed in glutamatergic (**c**) and GABAergic (**i**) afferent terminals and tdTomato expressed in STN neurons. tdTomato serves as a control fluorescent protein and a marker for STN to guide the probe implantation. **d**, **j**, Representative emission spectra of GCaMP6f expressed by glutamatergic (**d**) and GABAergic (**j**) axonal terminals and tdTomato expressed by STN neurons before and during DBS stimulation. **e**, **k**, Time-lapsed ratio of GCaMP6f/tdTomato fluorescence (normalized to the baseline before the first DBS stimulation) to show the DBS-induced changes in presynaptic Ca^2+^ levels in glutamatergic (**e**) and GABAergic (**k**) afferent terminals in the STN at different stimulation intensities. **f**, **l**, Summary of DBS-induced increases in presynaptic Ca^2+^ levels in glutamatergic (**f**) and GABAergic (**l**) afferent terminals in the STN at different stimulation intensities. **m**, **n**, Time-lapsed ratio of GCaMP6f/tdTomato fluorescence (normalized to the baseline before the first DBS stimulation) to show the DBS-induced changes in presynaptic Ca^2+^ levels in glutamatergic (**m**) and GABAergic (**n**) afferent terminals in the STN at the same stimulation intensity. * p < 0.05, ** p < 0.01 (For **f**: 100 µA vs. 150 µA, p = 0.0145; 150 µA vs. 200 µA, p = 0.0155; for **l**: 100 µA vs. 150 µA, p = 0.0028; 150 µA vs. 200 µA, p = 0.0064). Repeated one-way ANOVA followed by Dunnett’s multiple comparisons test. All data are plotted as mean ± SEM. n = 6 mice for the cortex group, n = 7 mice for GPe group, including both males and females. Source data

Consistent with the above findings, we observed similar robust activation in both glutamatergic and GABAergic axonal terminals during DBS in two additional cohorts of mice where we used different strategies, either by retrograde labeling using intra-STN injection of AAVretro-syn-jGCaMP7f-WPRE or by the combination of intra-cortical/intra-GPe injection of AAV9-hSyn-FlpO and intra-STN injection of AAV2retro-Ef1a-fDIO-GCaMP6f, to express GCaMP7f or GCaMP6f in afferent axon terminals innervating the STN (Extended Data Figs. 4 and 5).

### Effect of DBS on local neurotransmitter release

**Fig. 3:**
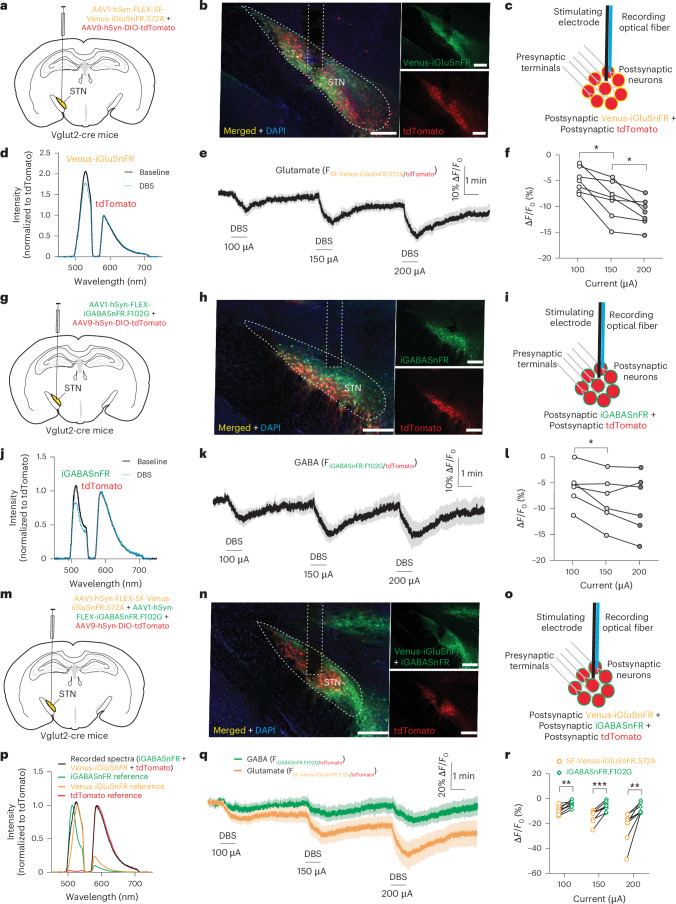
High-frequency STN DBS causes sustained inhibition in local neurotransmitter release in the STN of normal mice. **a**,**g**,**m**, Schematic illustrations showing microinjection of AAVs to express yellow glutamate sensor SF-Venus-iGluSnFR.S72A with tdTomato (**a**) and green GABA sensor iGABASnFR.F102G with tdTomato (**g**) and to co-express SF-Venus-iGluSnFR.S72A and iGABASnFR.F102G with tdTomato (**m**) in STN neurons. **b**,**h**,**n**, Representative images to show the expression of SF-Venus-iGluSnFR.S72A and tdTomato (**b**), iGABASnFR.F102G and tdTomato (**h**) and SF-Venus-iGluSnFR.S72A, iGABASnFR.F102G and tdTomato (**n**) in STN neurons. Scale bars, 200 μm. **c**,**i**,**o**, Illustrations of fiber photometry recordings of local glutamate release (**c**), GABA release (**i**) and simultaneous recording of glutamate and GABA release (**o**) using ratiometric fluorescence measurement of fluorescent sensors co-expressed with control fluorescent protein tdTomato. **d**,**j**, Representative emission spectra of SF-Venus-iGluSnFR.S72A and tdTomato (**d**) and iGABASnFR.F102G and tdTomato (**j**) expressed by STN neurons before and during DBS stimulation. **e**,**k**,**q**, Time-lapsed fluorescence ratio of SF-Venus-iGluSnFR.S72A/tdTomato (**e**), iGABASnFR.F102G/tdTomato (**k**) and SF-Venus- iGluSnFR.S72A/tdTomato and iGABASnFR.F102G/tdTomato recorded simultaneously from the same mice (**q**), normalized to the baseline before the first DBS stimulation, to show the DBS-induced changes in local glutamate and GABA release. **f**,**l**, Summary of DBS-induced changes in local glutamate (**f**) and GABA release (**l**) in the STN at different stimulation intensities. *P < 0.05 (for **f**: 100 µA versus 150 µA, *P* = 0.0186; 150 µA versus 200 µA, *P* = 0.0483; for **l**: 100 µA versus 150 µA, *P*= 0.0370), *n* = 7 mice for Venus-iGluSnFR group and *n* = 6 mice for iGABASnFR group, including both males and females, repeated one-way ANOVA followed by Dunnett’s multiple comparisons test. **p**, Representative emission spectrum (black) recorded from a mouse co-expressing SF-Venus-iGluSnFR.S72A, iGABASnFR.F102G and tdTomato in STN neurons (normalized by tdTomato emission peak), overlaid with normalized reference emission spectra of SF-Venus-iGluSnFR.S72A (yellow), iGABASnFR.F102G (green) and tdTomato (red). **r**, Comparison of DBS-induced changes between glutamate and GABA release at different stimulation intensities, recorded from mice co-expressing SF-Venus-iGluSnFR.S72A, iGABASnFR.F102G and tdTomato. **P < 0.01, ***P < 0.001 (100 µA: *P* = 0.0013, 150 µA: *P* = 0.0009, 200 µA: *P* = 0.0088), *n* = 8 mice, including both males and females, paired two-tailed *t*-test. Source data

**Extended Data Fig. 6:**
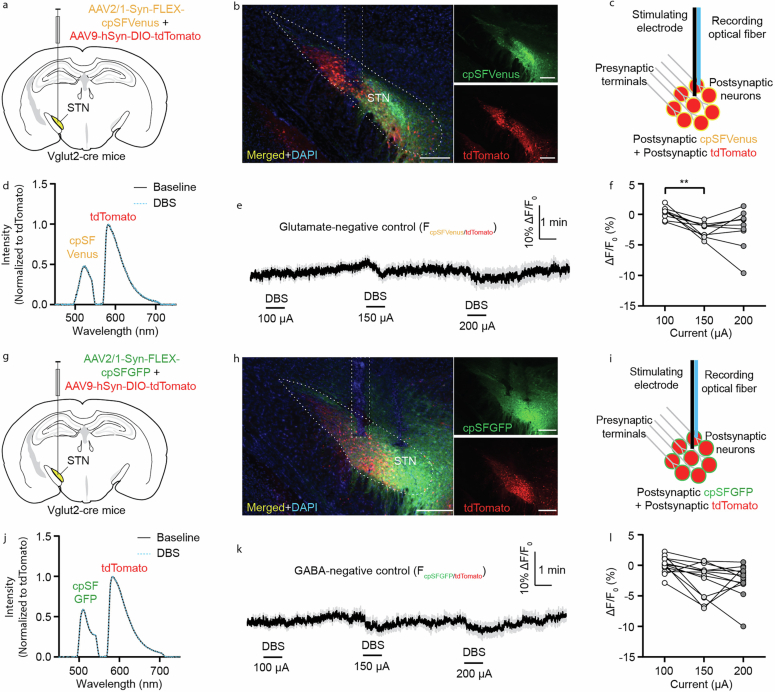
Effects of high-frequency STN DBS on negative controls of glutamate and GABA sensors in normal mice. **a**, **g**, Schematic illustrations showing the microinjection of AAVs to express cpSFVenus (negative control for SF-Venus-iGluSnFR) with tdTomato (**a**) and cpSFGFP (negative control for iGABASnFR) with tdTomato (**g**) in STN neurons. **b**, **h**, Representative images to show the expression of cpSFVenus and tdTomato (**b**) and cpSFGFP and tdTomato (**h**) in STN neurons. Scale bars: 200 μm. **c**, **i**, Illustrations of fiber photometry recordings of local cpSFVenus (**c**) and cpSFGFP (**i**) co-expressed with control fluorescent protein tdTomato. **d**, **j**, Representative emission spectra of cpSFVenus and tdTomato (**d**) and cpSFGFP and tdTomato (**j**) expressed by STN neurons before and during DBS stimulation. **e**, **k**, Time-lapsed fluorescence ratio of cpSFVenus /tdTomato (**e**) and cpSFGFP/tdTomato (**k**), normalized to the baseline before the first DBS stimulation, to show the DBS-induced changes at different stimulation intensities. **f**, **l**, Summary of DBS-induced changes in fluorescence ratio of cpSFVenus /tdTomato (**f**) and cpSFGFP/tdTomato (**l**) in the STN at different stimulation intensities. ** p < 0.01 (For **f**: 100 µA vs. 150 µA, p = 0.0019), Repeated one-way ANOVA followed by Dunnett’s multiple comparisons test. All data are plotted as mean ± SEM. n = 9 from 7 mice (two mice had bilateral implants), including both males and females, for cpSFVenus group; n = 13 from 7 mice (6 mice had bilateral implants), including both males and females, for cpSFGFP group. Source data

To investigate how DBS causes contrasting effects on presynaptic and postsynaptic components around the stimulation site, we examined DBS-induced changes in the levels of extracellular glutamate and GABA in the STN. To measure local glutamate release, we expressed yellow fluorescent glutamate sensor SF-Venus-iGluSnFR.S72A and red fluorescent control tdTomato in STN neurons by injecting AAV1-hSyn-FLEX-SF-Venus-iGluSnFR.S72A and AAV9-hSyn-DIO-tdTomato into the STN of Vglut2-cre mice and implanted the electrode−optical fiber hybrid probe into the STN (Fig. 3a−c). We found that DBS caused profound, stimulating, intensity-dependent inhibition in local glutamate release, measured by the percent change of the ratio of Venus-iGluSnFR/tdTomato fluorescence (F_Venus-iGluSnFR/tdTomato_) (100 µA: −4.857 ± 0.8920; 150 µA: −8.685 ± 1.395; 200 µA: −11.17 ± 1.022; Fig. 3d−f). To measure local GABA release, we expressed green fluorescent GABA sensor iGABASnFR.F102G and red fluorescent control tdTomato in STN neurons by injecting AAV1-hSyn-FLEX-iGABASnFR.F102G and AAV9-hSyn-DIO-tdTomato into the STN of Vglut2-cre mice and implanted the electrode−optical fiber hybrid probe into the STN (Fig. 3g−i). We found that, similar to the effect on glutamate release, DBS caused profound, stimulating, intensity-dependent inhibition in local GABA release, measured by the percent change of the ratio of iGABASnFR/tdTomato fluorescence (F_iGABASnFR/tdTomato_) (100 µA: −5.837 ± 1.494; 150 µA: −8.323 ± 1.896; 200 µA: −9.120 ± 2.354; Fig. 3j−l). To confirm that the observed changes in F_Venus-iGluSnFR/tdTomato_ and F_iGABASnFR/tdTomato_ were caused by the changes in extracellular glutamate and GABA, not by other factors that may affect the fluorescence, we repeated the above experiments, replacing the sensors with their corresponding parent fluorescent proteins cpSFVenus and cpSFGFP (Extended Data Fig. 6a−c,g−i). We found that DBS did not cause any changes in fluorescence at 100 µA but caused a small decrease in both the percent change of the ratio of F_cpSFVenus/tdTomato_ (100 µA: 0.2788 ± 0.3282; 150 µA: −2.596 ± 0.405; 200 µA: −2.413 ± 1.095; Fig. 3d−f and Extended Data Fig. 6d−f) and F_cpSFGFP/tdTomato_ (100 µA: 0.08273 ± 0.3723; 150 µA: −2.296 ± 0.7690; 200 µA: −2.466 ± 0.7415; Fig. 3j−l and Extended Data Fig. 6j−l) when stimulated at higher intensities, suggesting that a small fraction of DBS-induced decreases in F_Venus-iGluSnFR/tdTomato_ and F_iGABASnFR/tdTomato_ at higher stimulation intensities were caused by factors other than glutamate and GABA, possibly by local pH changes associated with the electrical stimulation^27^ or by local hemodynamic changes that may influence the fluorescence signals^28^.

**Extended Data Fig. 7:**
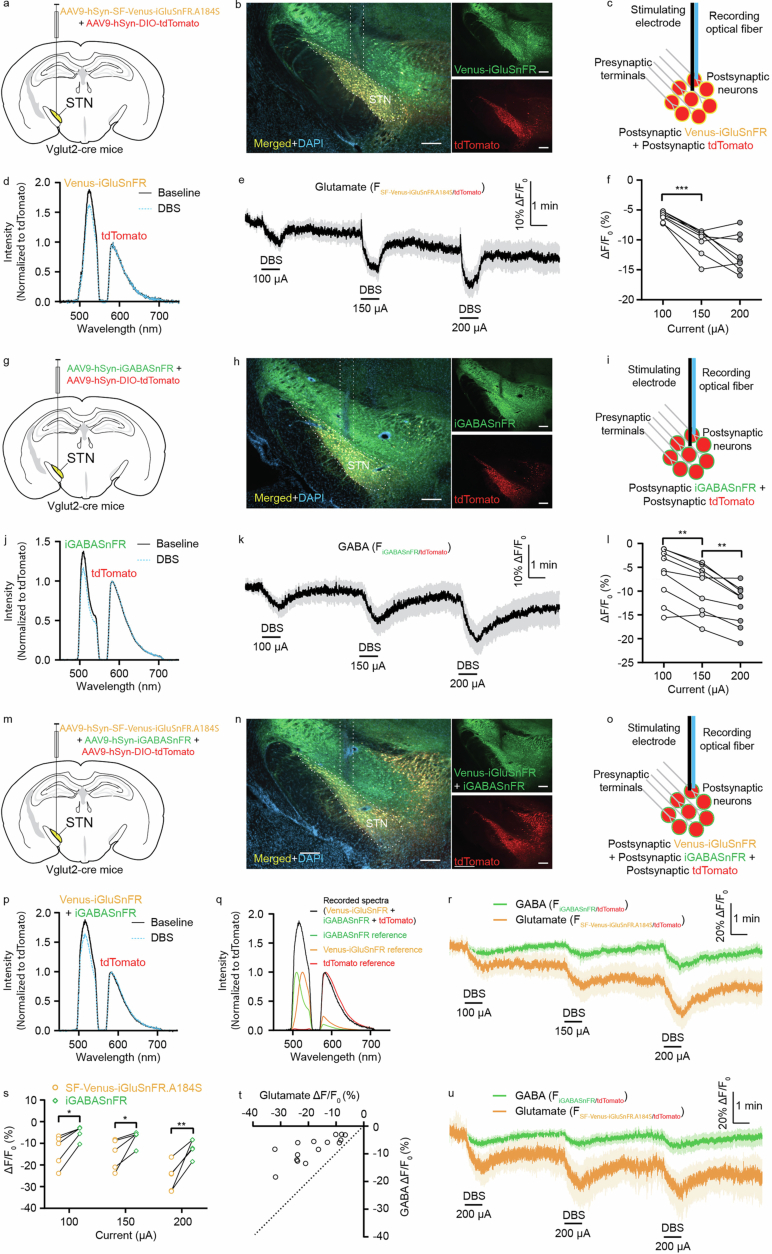
Validation of the effects of High-frequency STN DBS on local neurotransmitter release using a different pair of glutamate and GABA sensors in normal mice. **a**, **g**, **m**, Schematic illustrations showing the microinjection of AAVs to express yellow glutamate sensor SF-Venus-iGluSnFR-A184S with tdTomato (**a**), green GABA sensor iGABASnFR with tdTomato (**g**), and SF-Venus-iGluSnFR-A184S and iGABASnFR with tdTomato (**m**) in STN neurons. **b**, **h**, **n**, Representative images to show the expression of SF-Venus-iGluSnFR-A184S and tdTomato (**b**), iGABASnFR and tdTomato (**h**), and SF-Venus-iGluSnFR-A184S, iGABASnFR and tdTomato (**n**) in STN neurons. Scale bars: 200 μm. **c**, **i**, **o**, Illustrations of fiber photometry recordings of local glutamate release (**c**), GABA release (**i**), and simultaneous recording of glutamate and GABA release (**o**) using ratiometric fluorescence measurement of fluorescent sensors co-expressed with control fluorescent protein tdTomato. **d**, **j**, **p**, Representative emission spectra of SF-Venus-iGluSnFR-A184S and tdTomato (**d**) and iGABASnFR and tdTomato (**j**), and SF-Venus-iGluSnFR-A184S and iGABASnFR with tdTomato (**p**) expressed by STN neurons before and during DBS stimulation. **e**, **k**, Time-lapsed fluorescence ratio of SF-Venus-iGluSnFR-A184S/tdTomato (**e**) and iGABASnFR /tdTomato (**k**), normalized to the baseline before the first DBS stimulation, to show the DBS-induced changes in local glutamate and GABA release at different stimulation intensities. **f**, **l**, Summary of DBS-induced changes in local glutamate (**f**) and GABA release (**l**) in the STN at different stimulation intensities. ** p < 0.01, *** p < 0.001 (For **f**: 100 µA vs. 150 µA, p = 0.0003; for **l**: 100 µA vs. 150 µA, p = 0.0014; 150 µA vs. 200 µA, p = 0.0019), Repeated one-way ANOVA followed by Dunnett’s multiple comparisons test. **q**, Representative emission spectrum (black) recorded from a mouse co-expressing SF-Venus-iGluSnFR-A184S, iGABASnFR and tdTomato in STN neurons (normalized by tdTomato emission peak), overlaid with normalized reference emission spectra of SF-Venus-iGluSnFR-A184S (yellow), iGABASnFR (green) and tdTomato (red). **r**, **u**, Time-lapsed fluorescence ratio of SF-Venus-iGluSnFR-A184S/tdTomato and iGABASnFR /tdTomato recorded simultaneously from the same mice, normalized to the baseline before the first DBS stimulation, to show the DBS-induced changes in local glutamate and GABA release by the different stimulation intensities (**r**) and by the same stimulation intensity (**u**). **s**, **t**, Comparison of DBS-induced changes between glutamate and GABA release at different stimulation intensities, recorded from mice co-expressing SF-Venus-iGluSnFR-A184S, iGABASnFR and tdTomato. * p < 0.05, ** p < 0.01 (100 µA: p = 0.0145, 150 µA: p = 0.0469, 200 µA: p = 0.0069), Paired two tailed t test. All data are plotted as mean ± SEM. n = 8 mice for SF-Venus-iGluSnFR-A184S expressing group, n = 9 mice for iGABASnFR expressing group, n = 5 mice for SF-Venus-iGluSnFR-A184S and iGABASnFR co-expressing group, including both males and females. Source data

Because DBS inhibits both glutamate and GABA release in the STN, we next compared the magnitude of DBS-induced inhibition on glutamate and GABA release by simultaneously measuring glutamate and GABA release in the STN in the same mice during DBS. We injected the viral mixture of AAV1-hSyn-FLEX-SF-Venus-iGluSnFR.S72A, AAV1-hSyn-FLEX-iGABASnFR.F102G and AAV9-hSyn-DIO-tdTomato into the STN and implanted the electrode−optical fiber hybrid probe in the STN of Vglut2-cre mice (Fig. 3m−o). Using the linear unmixing feature that is unique to the spectrally resolved fiber photometry method^22^, we were able to unmix the closely overlapped emission spectra of SF-Venus-iGluSnFR.S72A and iGABASnFR.F102G (Fig. 3p) and used F_Venus-iGluSnFR/tdTomato_ and F_iGABASnFR/tdTomato_ to report extracellular glutamate and GABA levels, respectively, in the same mice. We found that, at all stimulating intensities, DBS causes a larger decrease in the extracellular level of glutamate than GABA (100 µA: ΔF_Venus-iGluSnFR/tdTomato_% = −9.302 ± 1.216, ΔF_iGABASnFR/tdTomato_% = −4.296 ± 0.9298; 150 µA: ΔF_Venus-iGluSnFR/tdTomato_% = −14.26 ± 1.880, ΔF_iGABASnFR/tdTomato_ % = −6.087 ± 1.363; 200 µA: ΔF_Venus-iGluSnFR/tdTomato_% = −22.19 ± 4.343, ΔF_iGABASnFR/tdTomato_ % = −8.104 ± 1.430; Fig. 3q,r), suggesting that DBS induces a shift in the local excitation/inhibition (E/I) ratio toward inhibition in the STN, which leads to suppressed neural activity in STN neurons during DBS. Because different versions of glutamate and GABA sensors have different sensitivities and dynamic ranges, which may affect the results during STN DBS, we repeated the above experiments using a different pair of sensors, SF-Venus-iGluSnFR.A184S and iGABASnFR, and came to the same conclusion (Extended Data Fig. 7).

### Effects of therapeutic DBS in parkinsonian mice

**Fig. 4:**
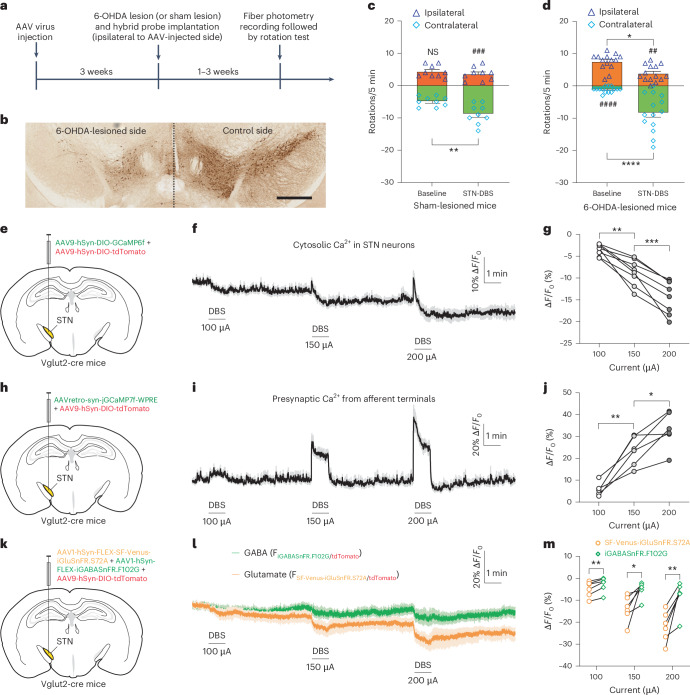
Effects of therapeutic STN DBS on intracellular Ca^2+^ levels in STN neurons and afferent presynaptic terminals and local glutamate and GABA release in unilateral 6-OHDA-lesioned PD mice. **a**, Timeline: unilateral AAV injection followed by ipsilateral 6-OHDA injection and hybrid probe implantation, fiber photometry recording and rotation tests in Vglut2-cre mice. **b**, DAB staining of TH^+^ cells in the midbrain of unilateral 6-OHDA-lesioned Vglut2-cre mice. Scale bars, 500 µm. **c**,**d**, Rotation tests to compare the number of spontaneous rotations before and during STN DBS (130 Hz, 60-µs pulse width, 150 µA) in sham-lesioned (**c**) and 6-OHDA-lesioned (**d**) Vglut2-cre mice. *P < 0.05, **P < 0.01, ****P < 0.0001, ^##^*P* < 0.01, ^###^*P* < 0.001, ^####^*P* < 0.0001 (for **c**: Baseline-Contralateral versus DBS-Contralateral, *P* = 0.0040; DBS-Ipsilateral versus DBS-Contralateral, *P* = 0.0001; for **d**: Baseline-Ipsilateral versus DBS-Ipsilateral, *P* = 0.0342; Baseline-Contralateral versus DBS-Contralateral, *P* < 0.0001; Baseline-Ipsilateral versus Baseline-Contralateral, *P* < 0.0001; DBS-Ipsilateral versus DBS-Contralateral, *P* = 0.0056), two-way ANOVA followed by Tukey’s multiple comparisons test. All data are plotted as mean ± s.e.m. *n* = 9 mice for sham-lesioned group, *n* = 14 mice for 6-OHDA-lesioned group, including both males and females. See Supplementary Videos 1 and 2 for representative behavioral videos. **e**,**h**,**k**, Schematic illustrations showing the microinjection of AAVs to express GCaMP6f with tdTomato in STN neurons (**e**), GCaMP7f in the afferent terminals in the STN and tdTomato in STN neurons (**h**) and SF-Venus-iGluSnFR.S72A, iGABASnFR.F102G and tdTomato in STN neurons (**k**). **f**,**i**,**l**, Time-lapsed fluorescence ratio (normalized to the baseline before the first DBS stimulation) of GCaMP6f/tdTomato in STN neurons (**f**), presynaptic GCaMP7f/postsynaptic tdTomato in the STN (**i**) and SF-Venus- iGluSnFR.S72A/tdTomato and iGABASnFR.F102G/tdTomato recorded simultaneously in the STN (**l**). **g**,**j**, Summary of DBS-induced changes in cytosolic Ca^2+^ levels in STN neurons (**g**) and afferent terminals in the STN (**j**) at different stimulation intensities. *P < 0.05, **P < 0.01, ***P < 0.001 (for **g**: 100 µA versus 150 µA, *P* = 0.0018; 150 µA versus 200 µA, *P* = 0.0006; for **j**: 100 µA versus 150 µA, *P* = 0.0032; 150 µA versus 200 µA, *P* = 0.0248), *n* = 8 mice for postsynaptic group (**g**) and *n* = 6 mice for presynaptic group (**j**), including both males and females, repeated one-way ANOVA followed by Dunnett’s multiple comparisons test. **m**, Comparison of DBS-induced changes between glutamate and GABA release at different stimulation intensities, recorded from mice co-expressing SF-Venus-iGluSnFR.S72A, iGABASnFR.F102G and tdTomato. *P < 0.05, **P < 0.01 (100 µA: *P* = 0.0028, 150 µA: *P* = 0.0282, 200 µA: *P* = 0.0083), *n* = 6 mice, including both males and females, paired two tailed *t*-test. NS, not significant. Source data

**Extended Data Fig. 8:**
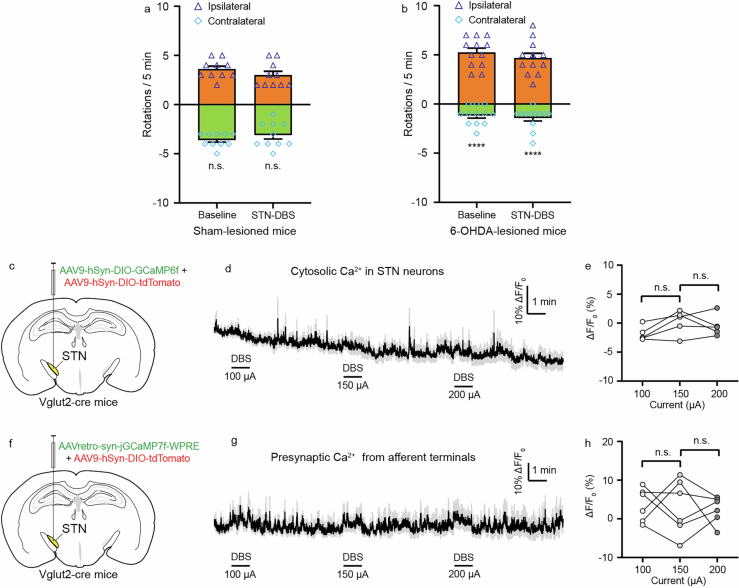
Lack of effect of low-frequency (20 Hz) unilateral STN DBS on behavior, intracellular Ca2+ levels in STN neurons and afferent presynaptic terminals in the STN of ipsilaterally 6-OHDA-lesioned mice. **a**, **b**, Rotation tests to compare the spontaneous rotations before and during low-frequency (20 Hz) STN DBS in sham-lesioned (**a**) and 6-OHDA-lesioned (**b**) Vglut2-cre mice. **** p < 0.0001 (For **b**: Baseline-Ipsilateral vs. Baseline-Contralateral, p < 0.0001; DBS-Ipsilateral vs. DBS-Contralateral, p < 0.0001), Two-way ANOVA followed by Tukey’s multiple comparisons test. All data are plotted as mean ± SEM. n = 10 mice for sham-lesioned group, n = 12 mice for 6-OHDA-lesioned group, including both males and females. **c**, **f**, Schematic illustrations showing the microinjection of AAVs to express GCaMP6f and tdTomato in STN neurons (**c**) and GCaMP7f in the afferent terminals in the STN and tdTomato in STN neurons (**f**). **d**, **g**, Time-lapsed fluorescence ratio (normalized to the baseline before the first DBS stimulation) of GCaMP6f/tdTomato in STN neurons (**d**) and presynaptic GCaMP7f/postsynaptic tdTomato in the STN (**g**) stimulated at different intensities with 20-Hz DBS. **e**, **h**, Summary of DBS-induced changes of the cytosolic Ca^2+^ in STN neurons (**e**) and afferent terminals in the STN (**h**) at different stimulation intensities with 20-Hz DBS. n = 5 mice for postsynaptic group (**e**) and n = 6 mice for presynaptic group (**h**), including both males and females, Repeated one-way ANOVA followed by Dunnett’s multiple comparisons test. Source data

To explore whether the DBS-induced changes that we observed in normal mice also occur in parkinsonian mice, we repeated the key experiments in a unilateral 6-hydroxy dopamine (6-OHDA) lesion hemi-parkinsonian model (Fig. 4a). We first injected AAVs to express the fluorescent sensors and tdTomato unilaterally in the STN of Vglut2-cre mice and then lesioned dopamine neurons in the same hemisphere by injecting 6-OHDA into the medial forebrain bundle (MFB) ipsilateral to the AAV-injected side (Fig. 4b) and implanted the electrode−optical fiber hybrid probe into the ipsilateral STN 3 weeks after the AAV injection. Fiber photometry recordings and rotation tests were carried out 1−3 weeks after the 6-OHDA lesion and probe implantation surgery. Unilateral DBS produced contralateral rotation bias in sham-lesioned mice (Fig. 4c) and corrected the spontaneous ipsilateral rotation bias in 6-OHDA-lesioned mice (Fig. 4d and Supplementary Videos 1 and 2), confirming that the DBS used in this study was therapeutic. In 6-OHDA-lesioned mice, we observed the same DBS-induced effects on STN neurons (Fig. 4e−g), afferent terminals (Fig. 4h−j) and glutamate and GABA levels (Fig. 4k−m) as we had observed in normal mice, suggesting that the mechanisms underlying DBS-induced behavioral effects are independent of the disease state. Next, we examined the frequency dependence of DBS-induced effects in these mice using 20 Hz for low-frequency DBS while keeping all other parameters unchanged. We found that low-frequency DBS did not induce a contralateral rotation bias in sham-lesioned mice and failed to correct the spontaneous ipsilateral rotation bias in 6-OHDA-lesioned mice (Extended Data Fig. 8a,b). Additionally, low-frequency DBS did not produce significant changes in intracellular Ca^2+^ levels in STN neurons or in the afferent presynaptic terminals within the STN (Extended Data Fig. 8c−h). These findings are consistent with previous reports showing that only high-frequency, not low-frequency, DBS is effective in treating PD^29,30^.

Based on the above results, we hypothesize that high-frequency DBS causes a persistent activation of the afferent fibers innervating STN neurons, which results in a decrease in neurotransmitter release, possibly due to the depletion of the vesicle pools, with a larger decrease in glutamate release than GABA. This shift of the local E/I ratio toward inhibition leads to a suppression of STN neurons during DBS.

**Extended Data Fig. 9:**
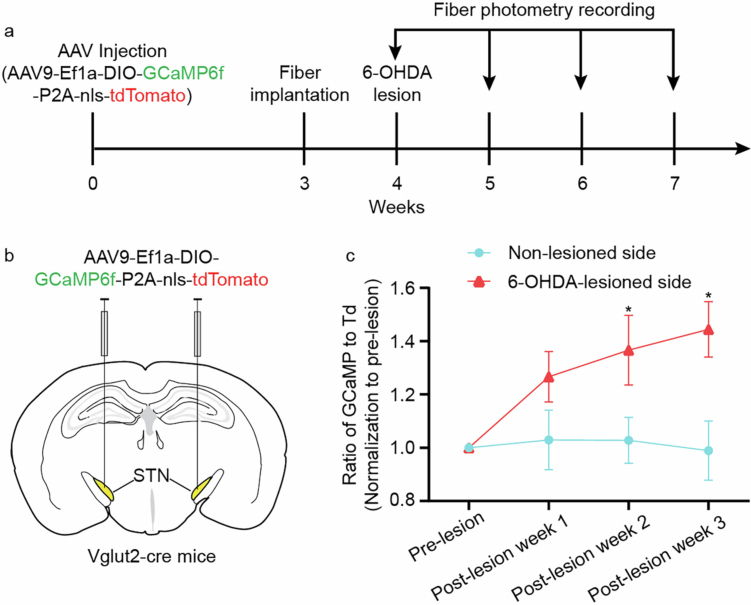
Effect of unilateral 6-OHDA lesion on intracellular Ca2+ levels in STN neurons. **a**, Timeline of the experiment: Bilateral AAV injections followed by bilateral fiber implantations in the STN, unilateral 6-OHDA injection into the MFB, and simultaneous bilateral (or, in some cases, unilateral, depending on the quality of GCaMP6f and tdTomato signals) fiber photometry recordings at one week before, and one, two, and three weeks after the 6-OHDA lesion in Vglut2-cre mice. **b**, Schematic illustration showing the microinjection of AAV to express GCaMP6f and tdTomato in STN neurons. **c**, Summary of unilateral 6-OHDA lesion-induced changes in intracellular Ca^2+^ levels in STN neurons on the lesioned and non-lesioned sides. * p < 0.05 (Post-lesion week 2, p = 0.0441; Post-lesion week 3, p = 0.0137), unpaired two tailed t-test. All data are plotted as mean ± SEM. n = 7, 6, and 6 for the post-lesion weeks 1, 2, and 3, respectively, on the 6-OHDA-lesioned side; n = 9, 8, and 8 for the post-lesion weeks 1, 2, and 3, respectively, on the non-lesioned side, from a total of 9 mice including both males and females. Source data

To investigate why DBS-induced inhibition of STN neurons was therapeutic in unilateral 6-OHDA-lesioned mice, we performed longitudinal bilateral fiber photometry recordings to monitor intracellular Ca^2+^ levels in STN neurons before and after unilateral 6-OHDA lesion in Vglut2-cre mice (Extended Data Fig. 9a,b). We observed that the 6-OHDA lesion caused a significant and sustained elevation of intracellular Ca^2+^ levels in STN neurons (Extended Data Fig. 9c). These results suggest that overall activity in STN neurons increases during PD^10,12^ and that DBS may be beneficial by counteracting the PD-induced rise in STN neuron activity^11,31^.

### Behavioral impact of chemogenetic modulation of STN in parkinsonian mice

**Fig. 5:**
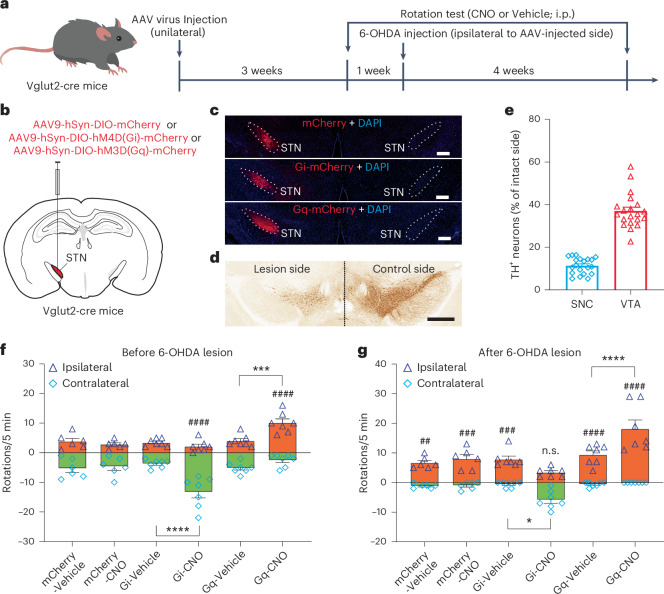
Chemogenetic inhibition of STN neurons restores motor function in 6-OHDA PD mice. **a**, Timeline for the virus/6-OHDA injection, drug treatments and behavioral tests in Vglut2-cre mice. **b**, Schematic illustration of unilateral viral delivery of AAV9-hSyn-DIO-hM4D(Gi)-mCherry, AAV9-hSyn-DIO-hM3D(Gq)-mCherry or AAV9-hSyn-DIO-mCherry to the STN of Vglut2-cre mice. **c**, Representative image to show the expression of mCherry, hM4D(Gi)-mCherry and hM3D(Gq)-mCherry in the unilateral STN of Vglut2-cre mice. Scale bars, 200 μm. **d**, DAB staining of TH^+^ neurons in the midbrain of Vglut2-cre mice after unilateral 6-OHDA lesion. Scale bar, 500 µm. **e**, Quantification of the change of TH^+^ neurons in the SNc and VTA of the lesion side compared to the control side in Vglut2-cre mice. **f**,**g**, Rotation tests to compare spontaneous turning behavior after the intraperitoneal injection of CNO or vehicle in Vglut2-cre mice expressing hM4D(Gi)-mCherry, hM3D(Gq)-mCherry or mCherry control unilaterally in the STN before (**f**) and after (**g**) ipsilateral 6-OHDA lesion. *P < 0.05, ***P < 0.001, ****P < 0.0001, ^##^*P* < 0.01, ^###^*P* < 0.001, ^####^*P* < 0.0001 (for **f**: Gi-Vehicle-Contralateral versus Gi-CNO-Contralateral, *P* < 0.0001; Gq-Vehicle-Ipsilateral versus Gq-CNO-Ipsilateral, *P* = 0.0009; Gi-CNO-Ipsilateral versus Gi-CNO-Contralateral, *P* < 0.0001; Gq-CNO-Ipsilateral versus Gq-CNO-Contralateral, *P* < 0.0001; for **g**: Gi-Vehicle-Contralateral versus Gi-CNO-Contralateral, *P* = 0.0259; Gq-Vehicle-Ipsilateral versus Gq-CNO-Ipsilateral, *P* < 0.0001; mCherry-Vehicle-Ipsilateral versus mCherry-Vehicle-Contralateral, *P* = 0.0057; mCherry-CNO-Ipsilateral versus mCherry-CNO-Contralateral, *P* = 0.0006; Gi-Vehicle-Ipsilateral versus Gi-Vehicle-Contralateral, *P* = 0.0002; Gq-Vehicle-Ipsilateral versus Gq-Vehicle-Contralateral, *P* < 0.0001; Gq-CNO-Ipsilateral versus Gq-CNO-Contralateral, *P* < 0.0001), two-way ANOVA followed by Tukey’s multiple comparisons test. All data are plotted as mean ± s.e.m. *n* = 6 mice for mCherry group, *n* = 7 mice for Gi-DREADD group, *n* = 7 mice for Gq-DREADD group, including both males and females. See Supplementary Videos 3 and 4 for representative behavior videos. i.p., intraperitoneal; VTA, ventral tegmental area. Source data

To further confirm that it is the inhibition, not excitation, of STN neurons that mediates the therapeutic effect of DBS, we compared the behavioral effects of chemogenetic inhibition versus excitation of STN neurons in the 6-OHDA-lesioned hemi-parkinsonian mice. We injected AAV9-hSyn-DIO-mCherry as control, inhibitory Gi Designer Receptor Exclusively Activated by Designer Drugs (DREADD) AAV9-hSyn-DIO-hM4D(Gi)-mCherry and excitatory Gq DREADD AAV9-hSyn-DIO-hM3D(Gq)-mCherry unilaterally in the STN of Vglut2-cre mice (Fig. 5a−c). Four weeks later, 6-OHDA was injected into the MFB ipsilateral to the STN that received virus injection to lesion midbrain dopamine neurons in the same hemisphere (Fig. 5a,d,e). Rotation tests were performed 1 week before and 4 weeks after the 6-OHDA injection. Before the 6-OHDA injection, DREADD agonist clozapine-N-oxide (CNO), 3 mg kg^−1^, injected intraperitoneally, did not induce spontaneous rotations in mice expressing mCherry in STN neurons (Vehicle: Contralateral = 5.333 ± 1.333, Ipsilateral = 3.833 ± 1.014; CNO: Contralateral = 4.667 ± 1.308, Ipsilateral = 2.833 ± 0.5426; Fig. 5f, left two columns). In mice expressing inhibitory Gi DREADD in the STN unilaterally, CNO administration resulted in a rotation bias toward the contralateral side (Vehicle: Contralateral = 3.714 ± 0.5216, Ipsilateral = 3.429 ± 0.4809; CNO: Contralateral = 13.29 ± 1.973, Ipsilateral = 2.143 ± 0.7377; Fig. 5f, center two columns). In mice expressing excitatory Gq DREADD in the STN unilaterally, CNO treatment induced an ipsilateral rotation bias (Vehicle: Contralateral = 5.143 ± 0.7047, Ipsilateral = 4.000 ± 0.7559; CNO: Contralateral = 2.571 ± 0.7825, Ipsilateral = 10.14 ± 1.299; Fig. 5f, right two columns). After the 6-OHDA lesion, all mice exhibited spontaneous ipsilateral rotations, which were not rescued by CNO in the mCherry control group (Vehicle: Contralateral = 1.167 ± 0.3073, Ipsilateral = 6.667 ± 0.8028; CNO: Contralateral = 1.000 ± 0.5164, Ipsilateral = 8.000 ± 1.438; Fig. 5g, left two columns). In the inhibitory Gi DREADD group, CNO fully rescued the deficits and restored balanced rotations (Vehicle: Contralateral = 0.5714 ± 0.3689, Ipsilateral = 7.857 ± 1.056; CNO: Contralateral = 5.857 ± 1.164, Ipsilateral = 3.429 ± 0.5714; Fig. 5g, center two columns, and Supplementary Video 3). In the excitatory Gq DREADD group, CNO treatment further exacerbated the symptoms and caused more biased ipsilateral rotations (Vehicle: Contralateral = 0.5714 ± 0.2974, Ipsilateral = 9.429 ± 1.288; CNO: Contralateral = 0, Ipsilateral = 18.14 ± 2.963; Fig. 5g, right two columns, and Supplementary Video 4). These results confirm that the inhibition, not excitation, of STN neurons is therapeutic for PD symptoms and inspired us to further explore whether the ‘chemogenetic DBS’—that is, chemogenetic inhibition of STN neurons using Gi DREADD—can become an effective treatment for advanced PD.

### Therapeutic effect of ‘chemogenetic DBS’ in MitoPark PD mice

**Fig. 6:**
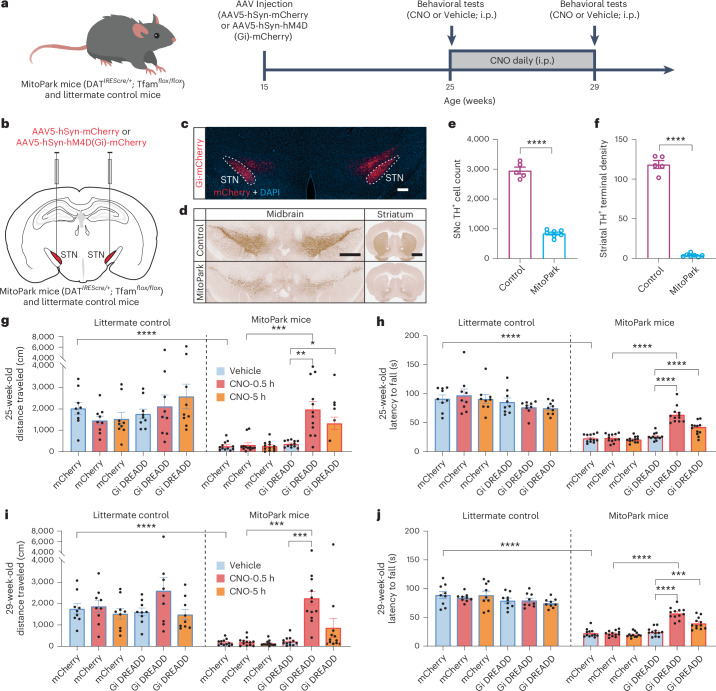
Chemogenetic inhibition of STN neurons restores motor function in a mouse model of advanced PD. **a**, Timeline for the virus injection, drug treatments and behavioral tests in MitoPark and littermate control mice. **b**, Schematic illustration of bilateral viral delivery of AAV5-hSyn-hM4D(Gi)-mCherry or AAV5-hSyn-mCherry to the STN of MitoPark and littermate control mice. **c**, Representative image to show the expression of hM4D(Gi)-mCherry in the STN of a MitoPark mouse. Scale bar, 200 μm. **d**, DAB staining of TH^+^ cells in the ventral midbrain and TH^+^ terminals in the striatum. Scale bars, 500 µm for midbrain and 1 mm for the striatum. **e**,**f**, Quantification of the number of TH^+^ neurons in the SNc (**e**) and the TH^+^ terminal optical density in the striatum (**f**) in 29-week-old MitoPark and littermate control mice. ****P < 0.0001 (for **e**, *P* < 0.0001; for **f**, *P* < 0.0001), unpaired two-tailed *t*-test. *n* = 5 mice for littermate control, *n* = 7 mice for MitoPark mice, including both males and females. **g**−**j**, Open field (**g**,**i**) and rotarod (**h**,**j**) tests in littermate control and MitoPark mice expressing hM4D(Gi)-mCherry or mCherry in the STN, measured longitudinally at the ages of 25 weeks (**g**,**h**) and 29 weeks (**i**,**j**). *P < 0.05, **P < 0.01, ***P < 0.001, ****P < 0.0001 (for **g**: Littermate-mCherry-Vehicle versus MitoPark-mCherry-Vehicle, *P* < 0.0001; MitoPark-mCherry-CNO-0.5 h versus MitoPark-Gi-CNO-0.5 h, *P*= 0.0008; MitoPark-Gi-Vehicle versus MitoPark-Gi-CNO-0.5 h, *P* = 0.0023; MitoPark-Gi-Vehicle versus MitoPark-Gi-CNO-5 h, *P* = 0.0144; for **h**: Littermate-mCherry-Vehicle versus MitoPark-mCherry-Vehicle, *P* < 0.0001; MitoPark-mCherry-CNO-0.5 h versus MitoPark-Gi-CNO-0.5 h, *P* < 0.0001; MitoPark-Gi-Vehicle versus MitoPark-Gi-CNO-0.5 h, *P* < 0.0001; MitoPark-Gi-Vehicle versus MitoPark-Gi-CNO-5 h, *P* < 0.0001; for **i**: Littermate-mCherry-Vehicle versus MitoPark-mCherry-Vehicle, *P* < 0.0001; MitoPark-mCherry-CNO-0.5 h versus MitoPark-Gi-CNO-0.5 h, *P* = 0.0002; MitoPark-Gi-Vehicle versus MitoPark-Gi-CNO-0.5 h, *P* = 0.0007; for **j**: Littermate-mCherry-Vehicle versus MitoPark-mCherry-Vehicle, *P* < 0.0001; MitoPark-mCherry-CNO-0.5 h versus MitoPark-Gi-CNO-0.5 h, *P* < 0.0001; MitoPark-Gi-Vehicle versus MitoPark-Gi-CNO-0.5 h, *P* < 0.0001; MitoPark-Gi-Vehicle versus MitoPark-Gi-CNO-5 h, *P* = 0.0009), two-way ANOVA followed by Tukey’s multiple comparisons test. All data are plotted as mean ± s.e.m. *n* = 9 mice for littermate control + mCherry group; *n* = 9 mice for littermate control + Gi-DREADD group; *n* = 12 mice for MitoPark + mCherry group; *n* = 12 mice for MitoPark + Gi-DREADD group, including both males and females. See Supplementary Video 5 for representative behavior videos. i.p., intraperitoneal. Source data

To further evaluate the therapeutic efficacy of the ‘chemogenetic DBS’ in a PD model that better resembles the symptoms in human patients, we used MitoPark mice^32^, a mouse PD model with a progressive loss of dopamine neurons and age-dependent motor deficits. We expressed Gi DREADD or mCherry control bilaterally in STN neurons by injecting AAV5-hSyn-hM4D(Gi)-mCherry or AAV5-hSyn-mCherry into the STN of MitoPark mice and their littermate controls when they were 15 weeks of age (Fig. 6a−c) and measured CNO-induced or vehicle-induced effects in open field and rotarod tests at the ages of 25 weeks and 29 weeks, when significant loss of dopamine neurons had occurred in the substantia nigra pars compacta (SNc dopamine neurons: littermate control = 2,954 ± 116 cells versus MitoPark = 842.1 ± 44.22 cells, counted from 11 sequential coronal sections; striatal TH^+^ terminal density: littermate control = 118.4 ± 4.936 versus MitoPark mice = 3.772 ± 0.7798, averaged from eight sequential striatal sections, measured at the age of 29 weeks; Fig. 6d−f and Supplementary Fig 1). At the age of 25 weeks, MitoPark mice already displayed severe motor deficits with reduced locomotor activity in open field tests (distance traveled: littermate control mice expressing mCherry after vehicle injection = 2,028 ± 288.1 cm; MitoPark mice expressing mCherry after vehicle injection = 258.9 ± 61.32 cm; Fig. 6g) and poor ability to maintain balance in rotarod tests (latency to fall: littermate control (in mice expressing mCherry control and after vehicle injection) = 91.24 ± 6.512 seconds; MitoPark (in mice expressing mCherry control and after vehicle injection) = 22.92 ± 1.950 seconds; Fig. 6h), resembling the symptoms observed in patients with advanced PD. However, a single dose of CNO, 3 mg kg^−1^ intraperitoneally, fully rescued the deficits in MitoPark mice expressing Gi DREADD in the STN in open field (distance traveled: Vehicle = 363.8 ± 44.12 cm, CNO = 2,564 ± 699.8 cm; Fig. 6g and Supplementary Video 5) and rotarod tests (latency to fall: Vehicle = 25.88 ± 1.789 seconds, CNO = 63.54 ± 4.099 seconds; Fig. 6h) within 30 minutes after the injection but did not cause behavioral effects in littermate controls. Furthermore, the therapeutic effect lasted for at least 5 hours (distance traveled in open field tests: Vehicle = 363.8 ± 44.12 cm, CNO = 1,328 ± 296.5 cm; latency to fall in rotarod tests: Vehicle = 25.88 ± 1.789 seconds, CNO = 42.54 ± 2.027 seconds; Fig. 6g,h).

Next, to evaluate the potential desensitization of the therapeutic effect of the ‘chemogenetic DBS’ after long-term treatment, mice received daily CNO, 3 mg kg^−1^ intraperitonally, for 4 weeks and were subjected to behavioral tests again at the age of 29 weeks before being euthanized for verification of virus expression in the STN. We found that 4 weeks of chronic CNO daily treatment did not cause desensitization in its therapeutic effect. The same dose of CNO, 3 mg kg^−1^ intraperitoneally, produced the same level of rescuing effect compared to its acute effect measured before the start of the chronic daily treatment in both open field tests (distance traveled: Vehicle = 230.6 ± 58.12 cm, CNO (measured 30 minutes after injection) = 2,136 ± 311.7 cm, CNO (measured 5 hours after injection) = 866.2 ± 443.6 cm; Fig. 6i) and rotarod tests (latency to fall: Vehicle = 24.61 ± 2.074 seconds, CNO (measured 30 minutes after injection) = 56.95 ± 2.942 seconds, CNO (measured 5 hours after injection) = 39.58 ± 2.957 seconds; Fig. 6j).

**Fig. 7:**
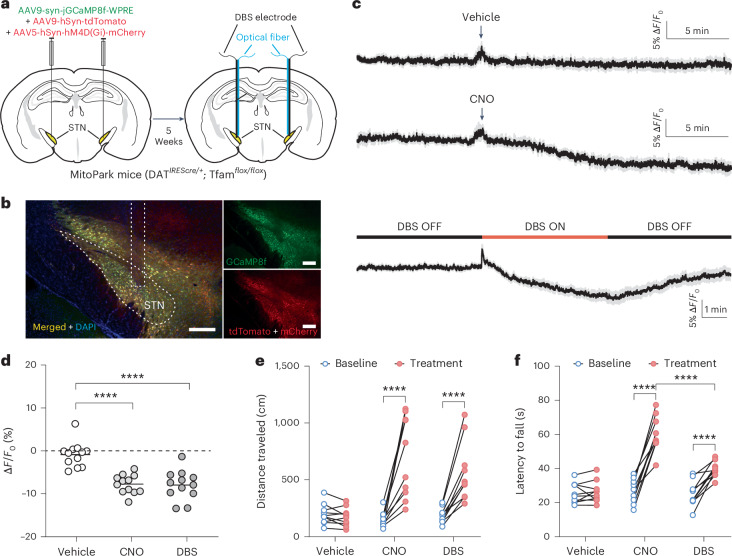
Comparison between chemogenetic and electrical DBS on rescuing motor deficits in MitoPark mice. **a**,**b**, Schematic illustration (**a**) and immunofluorescence staining (**b**) to show bilateral viral expression of GCaMP8f, tdTomato and hM4D(Gi)-mCherry in the STN followed by the implantation of DBS electrodes and optical fiber probes in MitoPark mice. Scale bar, 200 μm. **c**,**d**, Traces (**c**) and summary (**d**) of fiber photometry recordings of cytosolic Ca^2+^ in STN neurons, represented by % change of the fluorescence ratio of GCaMP8f/tdTomato, to show the effect of vehicle, CNO (3 mg kg^−1^) and electrical DBS (pulse width: 60 μs, intensity: 150 μA, frequency: 130 Hz) on STN neurons in MitoPark mice expressing GCaMP8f, tdTomato and hM4D(Gi)-mCherry in these neurons. ****P < 0.0001 (Vehicle versus CNO, *P* < 0.0001; Vehicle versus DBS, *P* < 0.0001), *n* = 12 mice, including both males and females, one-way ANOVA followed by Tukey’s multiple comparisons test. **e**,**f**, Open field (**e**) and rotarod (**f**) tests to show the effect of vehicle, CNO (3 mg kg^−1^) and electrical DBS (pulse width: 60 μs, intensity: 150 μA, frequency: 130 Hz) on the motor function of 25-week-old MitoPark mice. ****P < 0.0001 (for **e**: CNO, *P* < 0.0001; DBS, *P* < 0.0001; for **f**: CNO, *P* < 0.0001; DBS, *P* < 0.0001; CNO-Treatment versus DBS-Treatment, *P* < 0.0001), *n* = 10 mice, including both males and females, two-way ANOVA followed by Sidak’s multiple comparisons test. See Supplementary Video 6 for representative behavior videos. Source data

Finally, to directly compare the therapeutic efficacy between ‘chemogenetic DBS’ and electrical DBS, we expressed Gi DREADD, GCaMP8f and tdTomato bilaterally in the STN of MitoPark mice and implanted the electrode−optical fiber hybrid probes in the STN (Fig. 7a,b). We found that ‘chemogenetic DBS’, induced by an intraperitoneal injection of 3 mg kg^−1^ CNO, caused a similar inhibition in STN neurons, measured by the percent change of F_GCaMP8f/tdTomato_ (Vehicle: −0.8995 ± 0.8772; CNO: −8.031 ± 0.6596; DBS: −8.248 ± 1.020; Fig. 7c,d). The improvement of locomotor activity in the open field tests, measured by the travel distance (cm) in 10 minutes, was also similar between the chemogenetic and electrical DBS (for vehicle treatment: Baseline = 198.5 ± 28.11, Vehicle = 174.3 ± 25.31; for ‘chemogenetic DBS’: Baseline = 151.8 ± 20.95, CNO = 700.6 ± 112.4; for electrical DBS: Baseline = 184.2 ± 21.48, DBS = 564.9 ± 82.75; Fig. 7e and Supplementary Video 6), whereas the ‘chemogenetic DBS’ was more effective in rescuing motor deficits in rotarod tests than the electrical DBS (for vehicle treatment: Baseline = 25.61 ± 1.867 seconds, Vehicle = 26.25 ± 1.974 seconds; for ‘chemogenetic DBS’: Baseline = 26.99 ± 2.187 seconds, CNO = 60.28 ± 3.197 seconds; for electrical DBS: Baseline = 25.11 ± 2.320 seconds, DBS = 39.39 ± 1.415 seconds; Fig. 7f). These results suggest that this ‘chemogenetic DBS’ treatment can be equal to, or more effective than, treatment by electrical DBS.

## Discussion

In this study, we used genetically encoded fluorescent sensors to measure DBS-induced changes in presynaptic and postsynaptic Ca^2+^ and local neurotransmitter release in the STN. We found that therapeutic DBS causes sustained inhibition of local STN neurons in normal (Fig. 1), 6-OHDA-lesioned (Fig. 4f,g) and MitoPark (Fig. 7c,d) PD mice and that this inhibition extends to the axonal terminals in the SNr (Extended Data Fig. 3). These findings support the original hypothesis that DBS functions as reversible lesioning^2^. Our finding that Ca^2+^ in presynaptic terminals in the STN is persistently elevated during DBS (Figs. 2 and 4i,j) is consistent with previous findings that optical stimulation of afferent axonal terminals in the STN alleviates the symptoms in a 6-OHDA lesion PD model^18^ and that DBS excites cortical neurons that project to the STN^17^. However, we found that this persistent elevation of presynaptic Ca^2+^ does not translate to increased neurotransmitter release in the STN. Instead, both glutamate and GABA levels decreased during DBS (Figs. 3 and 4l,m), likely due to the depletion of vesicle pools after prolonged high-frequency stimulation^33^. The larger decrease in glutamate than GABA (Figs. 3m−r and 4k−m) indicates that high-frequency DBS shifts the local E/I ratio toward inhibition^34^, which explains the decreased neural activity in STN neurons during DBS. Although the exact mechanism underlying the differential depression of glutamatergic and GABAergic synapses during high-frequency stimulation is unclear, it is likely that vesicle pools in glutamatergic synapses are depleted at a faster rate than in GABAergic synapses under high-frequency stimulation, as similar observations were reported previously^35,36^ in the neocortex in slice preparation. Notably, the faster recovery of extracellular GABA than glutamate after the termination of DBS (Figs. 3q and 4l) is consistent with the difference in the vesicle recycling rate at glutamatergic and GABAergic synapses^35,37^. Although not directly tested in this study, it is plausible that the differential depression of glutamatergic and GABAergic synapses represents a general mechanism contributing to the therapeutic effects of DBS at other targets, such as the GPi and the ventral intermediate nucleus (Vim) of the thalamus. Although GPi and Vim neurons differ markedly from STN neurons in their intrinsic properties and circuit connectivity, they are under the dual control of both glutamatergic and GABAergic inputs. GABAergic synapses generally appear to be better adapted to high-frequency activity and exhibit greater resilience to repeated stimulation compared to glutamatergic synapses in the brain^35–38^.

It is important to note that, unlike presynaptic Ca^2+^, which rises instantly upon stimulation and returns to baseline immediately after DBS cessation (Figs. 2e,k and 4i), postsynaptic Ca^2+^ decreases gradually after DBS onset and remains reduced, recovering at a much slower rate (Figs. 1g and 4f). This slow onset of postsynaptic inhibition aligns with a progressive shift in the local E/I ratio, due to the differential depletion rates of glutamate and GABA synaptic vesicles. Similarly, the slow recovery is likely due to the prolonged recovery of the local E/I ratio, as glutamate recovers more slowly than GABA after DBS-induced vesicle depletion^35^, and it is common that the repeated stimulation-induced synaptic depression takes seconds to minutes to recover^39^. These differences in the timescales of presynaptic and postsynaptic responses suggest that DBS may exert its beneficial effects through multiple mechanisms. The immediate effect on tremor cessation corresponds with the rapid presynaptic response, whereas other benefits, such as alleviation of bradykinesia and rigidity, which take longer to manifest and do not cease immediately after DBS is turned off, are consistent with the slower kinetics of the postsynaptic response^40,41^. Interestingly, the effects of DBS on beta-band activity, which correlates with bradykinesia and rigidity, exhibit a slow onset and slow washout both at the initiation and cessation of therapeutic DBS^42^.

An advantage of using fiber photometry technique to investigate the mechanisms of DBS is that the recorded fluorescence signals are free of the large electrical stimulation artifacts seen in electrophysiological recordings during high-frequency DBS^43,44^. Another technical advance unique in this study is that, except for experiments shown in Fig. 7, we were monitoring the emission spectra of tdTomato and the fluorescent sensors expressed specifically by STN neurons and using the fluorescence signals to guide the placement of the hybrid probe during the implantation surgery. This photometry-guided surgery is critical for precise targeting of the STN in mice and increases the clinical relevance of this study, because it resembles the imaging-guided, electrophysiology-facilitated DBS surgeries in human patients and may account for some of the differences observed between this study and others^20,45^.

**Extended Data Fig. 10:**
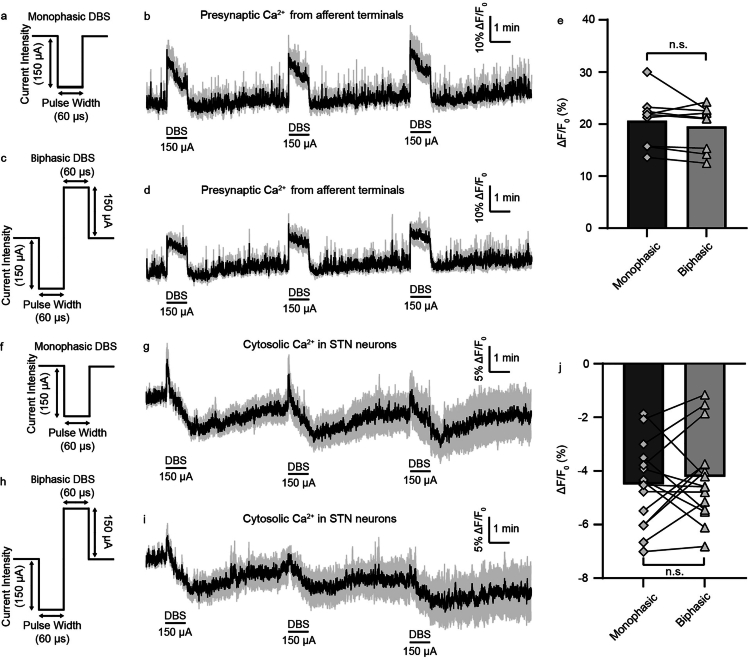
Comparison of the effects of monophasic and biphasic DBS on Ca²⁺ levels in afferent presynaptic terminals and in STN neurons of normal mice. **a**, **c**, **f**, **h**, Diagram showing the waveform of the stimulation pulses in monophasic DBS (**a**, **f**) and biphasic DBS (**c**, **h**). **b**, **d**, Time-lapsed ratio of GCaMP6f/tdTomato fluorescence (normalized to the baseline before the first DBS stimulation) to show the DBS-induced responses in presynaptic terminals in the STN with monophasic DBS (**b**) and biphasic DBS (**d**). **g**, **i**, Time-lapsed ratio of GCaMP6f/tdTomato fluorescence (normalized to the baseline before the first DBS stimulation) to show the DBS-induced responses in the STN neurons with monophasic DBS (**g**) and biphasic DBS (**i**). **e**, **j**, Summary of DBS-induced changes in presynaptic terminals in the STN (**e**) and in STN neurons (**j**). All data are plotted as mean ± SEM. n = 9 for presynaptic group from 3 mice with 3 repetitions (**e**), and n = 15 for postsynaptic group from 5 mice with 3 repetitions (**j**), including both males and females, unpaired two tailed t test. Source data

A previous study by Schor et al. reported that DBS elevated cytosolic Ca^2+^ levels in STN neurons^45^. The contrasting results between our study and theirs may be attributed to differences in electrode design and DBS settings. In our study, we used monopolar, monophasic cathodic stimulation, as this is the most commonly used stimulation mode in clinical practice^23^. By contrast, Schor et al. employed twisted-wire bipolar electrodes and biphasic stimulation pulses^45^. Because the cathodic and anodic stimuli exhibit different selectivity in activating axons and somata^46^, we tested whether the differences in pulse waveforms could account for the divergent results between our study and that of Schor et al. We compared DBS-induced changes in intracellular Ca^2+^ levels in STN neurons and afferent axonal terminals in the STN using either monophasic cathodic or biphasic pulses within the same group of mice. However, we did not find significant differences between the two stimulation waveforms (Extended Data Fig. 10). This leads us to think that the most likely explanation for the opposite results that we observed lies in the difference in electrode design and stimulation current intensity. With the same stimulation current intensity, monopolar electrodes activate a larger volume of tissue, resulting in a lower current density within the field, whereas bipolar electrodes create a more localized electrical field with a higher current density between the two closely placed electrodes^47^. In addition, they primarily used a stimulation current intensity of 200 µA in their study, whereas we used current intensities ranging from 100 µA to 200 µA. It is likely that the Ca^2+^ rise in STN neurons reported by Schor et al. was the result of membrane depolarization directly induced by strong electrical stimulation, bypassing the synaptic mechanisms. By contrast, the inhibition of STN neurons that we observed is likely synapse mediated, as axonal terminals can be excited at much lower current intensities compared to the soma^48^.

Taken together, intracellular Ca^2+^ levels in STN neurons, as a proximate indicator of neural activity, may either increase or decrease, depending on the DBS settings, especially the stimulation intensity. Stronger stimulations may depolarize the somata of STN neurons^45^ or even directly activate their axons, leading to a decoupling between the soma and axon^15,25^. Indeed, it was reported that neurons in the SNr, a major target of the STN, can be inhibited by low-intensity STN stimulation and excited by higher-intensity STN stimulation^16^. Additionally, STN DBS at 130 Hz, with a 60-µs pulse width and 500 µA, increases glutamate release in both the GP and SNr^49^.

A key question to ask when interpreting conflicting results is whether the DBS used in these studies produces therapeutic effects, which relates to another major difference between our study and that of Schor et al. In the unilateral 6-OHDA lesion PD model, we examined the rotation bias with and without the treatment^50^, whereas Schor et al. primarily used the movement speed as an indicator of the treatment efficacy. It should be noted that, in Schor et al., the spontaneous rotation bias in 6-OHDA-lesioned mice was only rescued by L-DOPA but not by their DBS. By contrast, in our study, both electrical and chemogenetic DBS successfully rescued the rotation bias in 6-OHDA-lesioned mice (Figs. 4d and 5g) and improved motor function in both open field and rotarod tests in MitoPark mice (Figs. 6 and 7e,f). Furthermore, if therapeutic DBS were to increase STN neural activity, as suggested by Schor et al., direct activation of STN neurons should theoretically be therapeutic. However, both our results and those of Xie et al.^21^ demonstrate that excitation of STN neurons exacerbates motor symptoms in 6-OHDA-lesioned animals (Fig. 5g).

Consistent with our findings that chemogenetic inhibition of STN neurons alleviates motor symptoms in 6-OHDA and MitoPark PD mice, pharmacological^51,52^ and lesional^11,53^ inhibition of STN promotes movement in normal animals and ameliorates motor symptoms in PD models and human patients. However, optogenetic modulation of STN neurons produces variable results in PD models^18,19,21^, likely due to differences in the neuronal populations targeted by the viral vectors and the coverage of the optical field. In contrast to our findings, where chemogenetic excitation of STN exacerbates motor symptoms in 6-OHDA-lesioned mice, McIver et al.^20^ demonstrated that chemogenetic activation of the STN ameliorates motor symptoms in 6-OHDA-lesioned mice. The discrepancies between our study and theirs may stem from differences in the viral spread of the AAV vectors and the behavioral assays used.

Compared to other modalities of antiparkinsonian interventions targeting the STN, such as local drug infusion^51,52^, lesioning^11,53^, DBS and optogenetic stimulation^18,19,21^, the chemogenetic gene therapy has its unique advantage as a reversible, less invasive and potentially more affordable alternative. Furthermore, the inhibitory and excitatory DREADDs, activated by different agonists^54^, can be targeted to multiple locations to achieve finely tuned network modulation. DREADD-mediated activation or inhibition in multiple brain regions has been shown to ameliorate motor deficits in the rodent and primate models of PD^55,56^.

To achieve specific chemogenetic inhibition of the STN neurons in the MitoPark PD model and potentially in humans, where the STN neurons do not express Cre or other recombinases to drive selective expression, we tested if we could achieve STN-specific expression of Gi DREADD in MitoPark mice by co-injection of cre-dependent Gi-DREADD AAV and AAV9-Vglut2-cre vectors into the STN. Unfortunately, unlike other studies reporting successful application of this strategy^57^, this approach did not produce desired specificity in our attempt (Supplementary Fig. 2). However, it remains hopeful that cell-specific targeting is plausible when more selective promoters, such as CaMKIIα promoter^18^, and RNA-based targeting strategies^58^ are used for targeting STN neurons.

It is important to note that our study focuses on the local mechanisms of therapeutic DBS within the STN. The sustained presynaptic activation of afferent terminals in the STN may trigger antidromic action potentials^17^, activating a broad cortical network to exert beneficial effects^1,6,7,25^. Furthermore, the DBS protocol used in this study is the conventional high-frequency STN DBS^2,23,24^. Other advanced DBS techniques, with different electrode design and stimulation patterns, may achieve therapeutic effects through distinct network-level mechanisms, depending on the specific input and output connections of the target site^59,60^. Additionally, although our findings support the idea that STN inhibition contributes to the therapeutic effects of DBS, they do not rule out the involvement of other mechanisms, such as modulation or disruption of PD-associated pathological activity, that may also play important roles^7,15,25^.

It is also important to note that the specific stimulation parameters used in DBS studies can substantially influence the observed outcomes. The stimulator used in this study does not support charge balancing in monophasic stimulation pulses. To achieve currents of 100–200 µA, the stimulation voltages ranged from approximately 1.5 V to 3 V and exhibited a reversible, intensity-dependent and duration-dependent voltage decline after the stimulation, suggesting that electrochemical changes occurred at the electrode–tissue interface. Although the absence of charge balancing is not ideal, its impact in this study may be minimal due to the relatively large electrode diameter and low stimulation intensities employed.

In summary, our results suggest that therapeutic high-frequency STN DBS inhibits STN neurons by differentially suppressing glutamatergic and GABAergic inputs, thereby shifting the local E/I ratio toward inhibition in the STN. The ‘chemogenetic DBS’—that is, chemogenetic inhibition of postsynaptic neurons—recapitulates the therapeutic effect of electrical DBS in the STN, pointing to a promising chemogenetic therapy that is less invasive and more affordable for patients with advanced PD or other refractory neuropsychiatric conditions.

## Methods

### Mice

All animal experiments were conducted as approved by the US National Institute of Environmental Health Sciences (NIEHS) Animal Care and Use Committee (ASP 2021-0014). MitoPark mice (DAT^*IREScre/+*^;Tfam^*flox/flox*^) were generated by two-step crossing between DAT^*IREScre/+*^ mice (The Jackson Laboratory, stock number 06660) and Tfam^*flox/flox*^ mice (The Jackson Laboratory, stock number 026123) as previously described^61^. Tfam^*flox/flox*^, Tfam^*flox/+*^ and DAT^*IREScre/+*^;Tfam^*flox/+*^ mice were used as littermate controls. Vglut2-cre mice were obtained from The Jackson Laboratory (stock number 028863) and bred in-house. All mice used in this study included both males and females aged 8−35 weeks and were housed under reverse light cycle conditions with access to food and water ad libitum at 18–23 °C with 40–60% humidity. Mice were group housed before receiving the cranial implants and singly housed afterwards.

### Viral vectors

AAV1-hSyn-FLEX-iGABASnFR.F102G (Addgene, 112164, titer: 2.4 × 10^13^ genome copies per milliliter (GC/ml)), AAV1-hSyn-FLEX-SF-Venus-iGluSnFR.S72A (Addgene, 106185, titer: 1.9 × 10^13^ GC/ml), AAV9-syn-jGCaMP8f-WPRE (Addgene, 162376, titer: 2.5 × 10^13^ GC/ml), AAV5-hSyn-mCherry (Addgene, 114472, titer: 1.4 × 10^13^ GC/ml), AAV5-hSyn-hM4D(Gi)-mCherry (Addgene, 50475, titer: 1.2 × 10^13^ GC/ml) and AAVretro-syn-jGCaMP7f-WPRE (Addgene, 104488, titer: 1.6 × 10^13^ GC/ml) were purchased from Addgene. AAV9-hSyn-iGABASnFR (Addgene, 112159, titer: 6.3 × 10^13^ GC/ml) and AAV9-hSyn-SF-Venus-iGluSnFR-A184S (Addgene, 106177, titer: 4.2 × 10^13^ GC/ml) were packaged by Vigene. AAV2/1-Syn-FLEX-cpSFGFP (titer: 3.7 × 10^13^ GC/ml) and AAV2/1-Syn-FLEX-cpSFVenus (titer: 7.3 × 10^13^ GC/ml) were gifts from Jonathan Marvin of the Howard Hughes Medical Institute Janelia Research Campus. AAV9-hSyn-DIO-GCaMP6f-WPRE (Addgene, 100833, titer: 2.9 × 10^13^ GC/ml), AAV9-hSyn-tdTomato (Addgene, 51506, titer: 1 × 10^13^ GC/ml), AAV9-Ef1a-DIO-GCaMP6f-P2A-nls-tdTomato (Addgene, 51083, titer: 6.0 × 10^11^ GC/ml), AAV9-hSyn-DIO-mCherry (Addgene, 50459, titer: 1.7 × 10^12^ GC/ml), AAV9-hSyn-DIO-hM4D(Gi)-mCherry (Addgene, 44362, titer: 8.5 × 10^12^ GC/ml), AAV9-hSyn-DIO-hM3D(Gq)-mCherry (Addgene, 44361, titer: 8 × 10^12^ GC/ml), AAV2retro-Ef1a-fDIO-GCaMP6f (a gift from Patricia Jensen of the NIEHS, titer: 9.4 × 10^12^ GC/ml), AAV9-hSyn-DIO-tdTomato (Addgene, 51505, titer: 2 × 10^13^ GC/ml), AAV9-hSyn-FlpO (Addgene, 60663, titer: 1.4 × 10^14^ GC/ml) and AAV9-Vglut2-cre (titer: 9.46 × 10^11^ GC/ml) were packaged by triple plasmid transfection of HEK293 cells (Agilent, 240073, AAV Helper-Free System) by the NIEHS Viral Vector Core.

To create vector DNA pAAV-vGluT2-cre, *Mus musculus* vesicular glutamate transporter 2 (vGluT2, sSlc17a6) gene promoter was subcloned according to GenBank sequences (DQ812098.1). The polymerase chain reaction (PCR) forward primer was 5’-TTGCACGCGTGG GCCCACGCACTC CCCTGGTTGATT TAGCTAGCATCC-3’ and PCR primer reverse: 5′-GCCATCTAGAG CGGCCGCTCTTG TAAAGACTGGTG TCCAGCCTTACC AGATTTAAATTG-3’. Then, the PCR-amplified vGluT2 promoter sequence was inserted into the pAAV-Cre backbone.

### Stereotaxic microinjections of AAVs

AAVs were injected at the rate of 10−50 nl min^−1^ through a Hamilton Neuros syringe with a 30-gauge needle via standard stereotaxic procedures with mice under isoflurane anesthesia^62^. The needle was left in place for 10 more minutes before withdrawal. The coordinates used for targeting the STN were AP: −2.0 mm, ML: ±1.60 mm from bregma and DV: −4.6 mm from the brain surface.

For experiments measuring postsynaptic Ca^2+^, extracellular glutamate and extracellular GABA and for experiments measuring extracellular glutamate and GABA simultaneously in the STN, the following viral mixtures were injected into the STN, respectively. For postsynaptic Ca^2+^: AAV9-hSyn-DIO-GCaMP6f-WPRE and AAV9-hSyn-DIO-tdTomato (volume ratio 2:1; total volume = 150 nl); for extracellular glutamate: AAV1-hSyn-FLEX-SF-Venus-iGluSnFR.S72A and AAV9-hSyn-DIO-tdTomato (volume ratio 2:1; total volume = 150 nl); for extracellular GABA: AAV1-hSyn-FLEX-iGABASnFR.F102G and AAV9-hSyn-DIO-tdTomato (volume ratio 2:1; total volume = 150 nl); and for simultaneous glutamate and GABA: AAV1-hSyn-FLEX-SF-Venus-iGluSnFR.S72A, AAV1-hSyn-FLEX-iGABASnFR.F102G and AAV9-hSyn-DIO-tdTomato (volume ratio 1:2:1; total volume = 150 nl). For longitudinal monitoring of STN neuronal activity before and after unilateral 6-OHDA lesion (Extended Data Fig. 9), 150 nl of AAV9-Ef1a-DIO-GCaMP6f-P2A-nls-tdTomato was injected bilaterally into the STN of Vglut2-cre mice.

To record the presynaptic Ca^2+^ from the glutamatergic and GABAergic afferent terminals in the STN, AAV9-hSyn-GCaMP8f was injected into the M1 motor cortex at two locations (AP: +0.5 mm and 1.5 mm, ML: ±1.25 mm from bregma, DV: −1.1 mm from the brain surface; volume = 50 nl per site) or into the GPe (AP: +0 mm, ML: ±1.5 mm from bregma, DV: −3.8 mm from the brain surface; volume = 50 nl per site); AAV9-hSyn-DIO-tdTomato (200 nl) was injected into the STN of Vglut2-cre mice. To record the presynaptic Ca^2+^ from the glutamatergic and GABAergic afferent terminals in the STN using a different strategy (Extended Data Fig. 5), AAV9-hSyn-FlpO was injected into the M1 motor cortex or into the GPe following the same procedure as described above, followed by a second injection of AAV2retro-Ef1a-fDIO-GCaMP6f and AAV9-hSyn-DIO-tdTomato (volume ratio 5:1; total volume = 200 nl) into the STN of Vglut2-cre mice 3 weeks later. To record the presynaptic Ca^2+^ non-selectively from both glutamatergic and GABAergic afferent terminals in the STN (Extended Data Fig. 4), AAVretro-syn-jGCaMP7f-WPRE and AAV9-hSyn-DIO-tdTomato (volume ratio 5:1; total volume = 200 nl) were injected into the STN.

To evaluate the therapeutic effect of ‘chemogenetic DBS’ on motor function in MitoPark mice, AAV5-hSyn-mCherry (300 nl) or AAV5-hSyn-hM4D(Gi)-mCherry (300 nl) was micro-injected bilaterally into the STN of MitoPark and littermate control mice. To compare the effects of electrical DBS and ‘chemogenetic DBS’ on STN neural activity and motor function, MitoPark mice first received a bilateral intra-STN injection of AAV5-hSyn-hM4D(Gi)-mCherry (200 nl) and then a second bilateral intra-STN injection of the viral mixture containing AAV9-syn-GCaMP8f-WPRE and AAV9-hSyn-tdTomato (volume ratio 2:1; total volume = 200 nl) 2−3 weeks later. For chemogenetic inhibition or excitation of the STN neurons in the 6-OHDA PD model, AAV9-hSyn-DIO-mCherry (30 nl), AAV9-hSyn-DIO-hM4D(Gi)-mCherry (30 nl) or AAV9-hSyn-DIO-hM3D(Gq)-mCherry (30 nl) was micro-injected into the STN ipsilateral to the 6-OHDA-lesioned side of Vglut2-cre mice.

### Construction of optical fiber−electrode hybrid probes

The hybrid probes were constructed by combining an optical fiber probe and a tungsten electrode together. The optical fiber probes were constructed from a piece of multimode optical fiber (core diameter: 105 µm, cladding diameter: 125 µm, numerical aperture: 0.22; Thorlabs, FG105LCA) and a ceramic ferule (diameter: 1.25 mm) from a multimode Lucent connector (Precision Fiber Products, MM-CON2007). The optical probes were first polished on the ferrule end and then flat-cleaved on the fiber end to have a 7-mm fiber length. The tungsten electrodes were fabricated by soldering a piece of perfluoroalkoxy-coated tungsten wire (bare wire diameter: 127 µm, diameter with insulation: 178 µm; A-M Systems, catalogue number 796500) to a male connector pin (A-M Systems, catalogue number 520200). The tungsten wire was bent at two points to allow the straight section of the wire to align well with the optical fiber and cut to 7 mm long. Under a dissection microscope, the optical fiber probe and the tungsten electrode were placed in parallel and in contact, with the end of the tungsten wire protruding 0.1−0.2 mm relative to the end of the optical fiber and then bound together by C&B Metabond (Parkell).

### Fiber photometry-guided surgery for optical fiber−electrode hybrid probe and optical fiber probe implantation

Three to four weeks after the virus injection, mice were subjected to the second stereotaxic surgery for hybrid probe or optical fiber probe implantation following a similar procedure as previously described^22^. For implantation of optical fiber−electrode hybrid probes targeting the STN, two burr holes were drilled through the skull above the STN bilaterally (AP: −2.0 mm, ML: ±1.60 mm from bregma). Three more burr holes were drilled on the skull for anchoring screws. After the anchoring screws were put in place, a brain surface electrode, constructed from a piece of uncoated stainless steel wire (diameter: 203 µm; A-M Systems, catalogue number 792900) and a male connector pin (A-M Systems, catalogue number 520200), was tightly wrapped around each screw to ensure good conductivity. Then, the spectrally resolved fiber photometry system was powered on^22^. The output power of the 488-nm laser was set at 50−75 µW, measured at the end of the patch cable. Next, the fiber−electrode hybrid probe was connected to the patch cable. In the spectrometer operation software OceanView (Ocean Optics), the background spectrum detected by the spectrometer was subtracted when the room light was turned off. The hybrid probe was lowered above the brain surface through the burr hole and then further lowered toward the STN until the emission spectrum of tdTomato or other cre-dependent fluorescent proteins started to appear in OceanView. The probe was then lowered slowly while the magnitude of the emission spectrum was being monitored until it reached a plateau. The final hybrid probe tip location was approximately 4.5−4.6 mm below the brain surface. The probe was then fixed in place with a generous amount of dental acrylic (Jet; Lang Dental Manufacturing Co.). The mice were then singly housed and allowed 1 week to recover before experiments proceeded.

For experiments where STN fiber photometry recordings were performed without DBS (Extended Data Fig. 9), optical fiber probes were implanted following a procedure similar to the one described above, with the exception that the hybrid probes were replaced with optical fiber probes, and the brain surface electrode was not implanted.

In experiments where fiber photometry recordings were conducted from the axonal terminals of STN neurons in the SNr during STN DBS (Extended Data Fig. 3), two additional burr holes were drilled bilaterally above the SNr (AP: −3.2 mm, ML: ±2.65 mm from bregma). The optical fiber probes were then lowered toward the SNr at a 15° medial angle to create more space between the ferrules of the two probes. The final position of the probe tip was approximately 4.2–4.4 mm from the entry point on the brain surface.

### Spectrally resolved fiber photometry during DBS

The fiber photometry recordings were conducted using a lab-built spectrally resolved fiber photometry system^22^ (https://www.niehs.nih.gov/research/atniehs/labs/ln/pi/iv/tools) paired with genetically encoded fluorescent sensors^63–67^ to target presynaptic and postsynaptic components and neurotransmitters in the STN and carried out in freely moving mice in an open-top mouse operant chamber (21.6 cm × 17.8 cm × 12.7 cm; Med Associates, ENV-307W-CT) housed in a sound-attenuating box (Med Associates, ENV-017M). For fiber photometry recordings in 6-OHDA-lesioned mice, the mice were placed on a low-profile mouse running wheel (Med Associates, ENV-047) in the chamber to reduce the fiber and cable twisting. Fluorescence spectra were acquired by a CMOS-based modular spectrometer (Ocean Optics, Ocean FX) using 30 -ms integration time, triggered by 25-Hz transistor−transistor logic (TTL) pulses sent from a digital output module (Med Associates, DIG-726TTL) on a customized mouse operant conditioning package (Med Associates). The output power of the 488-nm laser measured at the end of the patch cable was set at approximately 50−75 µW. A digital video camera (FLIR Integrated Imaging Solutions, Grasshopper3 GS3-U3-23S6M-C), frame-by-frame triggered by the same TTL pulses triggering the spectrometer, was used to record the animal’s behavior. One minute after the start of the fiber photometry recording, a train of electrical stimulating pulses (130 Hz, 60-μs pulse width, 60 seconds long) (Extended Data Fig. 1) with three current intensities (100 µA, 150 µA and 200 µA) were delivered with 5-minute intervals between each train using a constant-current isolated stimulator (A-M Systems, model 2100) controlled by a waveform generator (Siglent Technologies, SDG1025). The output voltage during constant-current stimulation was monitored using an oscilloscope (Tektronix, TBS 1102B-EDU) with a high-impedance probe (Tektronix, TPP0101), connected across the two output terminals of the stimulator. For the low-frequency DBS experiments (Extended Data Fig. 8), 20-Hz stimulation pulses were applied, with all other parameters kept the same. For experiments comparing monophasic and biphasic DBS stimulation (Extended Data Fig. 10), either monophasic (Extended Data Fig. 10a,f) or biphasic (Extended Data Fig. 10c,h) pulses with a 60-μs pulse width were delivered at 130 Hz, with a current intensity of 150 µA. Spectral linear unmixing^22^ was performed on the acquired fluorescence emission spectra to isolate individual spectral components using a customized program written in R (https://www.niehs.nih.gov/research/atniehs/labs/ln/pi/iv/tools). To correct for the fluorescence fading, we first applied linear regression to fit the first 1 minute of the unmixed coefficients plotted over time and then used the fitted curve as the theoretical baseline (*F*_0_) to calculate Δ*F*/*F*_0_%.

### Unilateral 6-OHDA lesion in Vglut2-cre mice

6-OHDA (Sigma-Aldrich, H4381) was dissolved in sterile 0.9% saline containing 0.02% ascorbic acid to make 3 mg ml^−1^ solution, kept on ice, protected from light and used within 4 hours after preparation. Vglut2-cre mice received an intraperitoneal injection of desipramine hydrochloride (25 mg kg^−1^) and pargyline hydrochloride (5 mg kg^−1^) 30 minutes before the 6-OHDA injection. Then, 6-OHDA (1.5 µg in 0.5 µl total volume) was injected at 0.1 µl min^−1^ unilaterally (counterbalanced between the left and right hemispheres) through a Hamilton Neuros syringe with a 30-gauge needle into the MFB (AP: −1.20 mm, ML: ±1.25 mm from bregma and DV: −4.80 mm from the brain surface). For the sham lesion, an empty Hamilton Neuros syringe with a 30-gauge needle was lowered into the brain to target the MFB and left in place for 5 minutes before being withdrawn.

### Behavioral tests

For the unilateral 6-OHDA lesion PD model, cylinder rotation tests were conducted before or 3−4 weeks after the unilateral injection of 6-OHDA into the MFB of Vglut2-cre mice. The mice were individually placed in a glass cylinder (diameter: 13 cm, height: 20 cm) inside a sound-attenuating chamber with the lights off, and videos were recorded by an infrared camera mounted on the ceiling. For mice receiving DBS treatment, they were placed in the cylinder and recorded for 15 minutes. The first 5 minutes had DBS off, followed by 5 minutes with DBS on and the final 5 minutes with DBS off again. For mice receiving chemogenetic treatment, on the first day the mice were randomly assigned to vehicle or CNO groups. Thirty minutes after intraperitoneal injection of either CNO or vehicle, the mice were placed in the cylinder and recorded for 5 minutes. On the 2nd day, the treatment groups were switched, and the rotation test was repeated.

For MitoPark and littermate control mice, the behavioral tests were first conducted when the mice were 25 weeks old, 10 weeks after the virus injection to express Gi DREADD or mCherry control in STN neurons. CNO was first dissolved in DMSO to make 60 mg ml^−1^ stock solution. Then, 5 µl of stock solution was dissolved in 1 ml of sterile 0.9% saline to make the final injection solution. CNO (3 mg kg^−1^) or equal volume of 0.5% DMSO vehicle control was intraperitoneally injected into the mice 30 minutes before tests started. Behavioral tests were repeated at 5 hours after CNO or vehicle injection. Then, daily intraperitoneal injections of CNO were conducted for 4 weeks, and motor functions of the mice were assessed again at 29 weeks of age.

Open field tests were performed for checking the general locomotor activity, and rotarod tests were performed for checking the motor skills and coordination. In open field tests, the MitoPark and their littermate control mice were individually placed in the center of the arena (27.31 cm × 27.31 cm × 20.32 cm; Med Associates, ENV-510S) and monitored for 30 minutes. The movements of the mice were tracked and analyzed using Med Activity Monitor software (Med Associates). On the first day, mice were placed in the arena for 30 minutes for acclimation to the environment. On the 2nd day, half of the mice received an intraperitoneal injection of vehicle, and the other half received an intraperitoneal injection of CNO. Thirty minutes after the injections, the movements of the mice were recorded for another 30 minutes. The open field tests were repeated at 5 hours after CNO or vehicle injection. No tests were performed on the 3rd day. On the 4th day, the open field tests were repeated with vehicle-treated and CNO-treated mice on day 2 receiving CNO and vehicle, respectively. The total travel distance was calculated for the evaluation of locomotor activity.

For rotarod tests, MitoPark and littermate control mice were first pre-trained on an automated six-lane rotarod for three consecutive days, with one training session per day, each session consisting of three trials separated by a 30-minute interval in between. In each trial, the rotarod started at 4 revolutions per minute (r/min) and accelerated to 40 r/min with an acceleration of 20 r/min^2^. The trial ended when the mice fell off the rod or started cartwheeling or when the trial time reached 5 minutes. On the day after the last pre-training day, mice were tested in three trials. The latency to fall, averaged across three trials, was used to evaluate the motor skills and coordination.

### Comparing the therapeutic effects between ‘chemogenetic DBS’ and electrical DBS

To compare the effects of ‘chemogenetic DBS’ and electrical DBS on the activity of STN neurons in MitoPark mice, fiber photometry recordings were conducted while the mice received a train of electrical stimulating pulses (130 Hz, 60-μs pulse width) with current intensity of 150 µA for 5 minutes after a 5-minute baseline. The recording continued for another 5 minutes after the cessation of the electrical DBS. Two days later, these mice underwent another session of fiber photometry recording for 30 minutes, with half of the mice receiving an intraperitoneal injection of CNO (3 mg kg^−1^) and half of the mice receiving vehicle injection 10 minutes after the start of the recording. The next day, the mice underwent another session of fiber photometry recording for 30 minutes with the vehicle and CNO treatment reversed from the day before.

For open field tests, on the first day mice were kept in the arena for 35 minutes to acclimate to the environment. On the 2nd, 4th and 6th days, mice received vehicle injection, CNO injection (3 mg kg^−1^) or electrical DBS treatment (130 Hz, 60-μs pulse width, 150 µA, 20-minute total duration) at 15 minutes after the start of the open field test and continued to be monitored in the arena for another 20 minutes. The activity between the 5th and 15th minute was used as the baseline. The activity between the 25th and the 35th minute was used to compare the effects of vehicle, CNO and electrical DBS.

For rotarod tests, mice were pre-trained on the rotarod for three consecutive days. On the 4th, 6th and 8th days, mice received vehicle injection, CNO injection (3 mg kg^−1^) or electrical DBS treatment (130 Hz, 60-μs pulse width, 150 µA). Vehicle or CNO was given 30 minutes before the rotarod test. For electrical DBS treatment, the stimulation started 10 minutes before the test and continued until the mice fell off the rotarod.

To ensure fair comparison, the mice were connected to the electrical cables in all the experiments described in this section regardless of whether the electrical DBS was given.

### Immunofluorescence/immunohistochemistry

Mice were transcardially perfused with 20 ml of ice-cold PBS followed by 20 ml of 4% paraformaldehyde (PFA). Brains were post-fixed in 4% PFA overnight and then transferred to 30% sucrose (made with PBS) for storage at 4 °C until further processing. Coronal slices were sectioned on a microtome (Thermo Fisher Scientific, KS34) at a thickness of 35 µm. For immunofluorescence staining, sections were blocked with 10% normal goat serum (Vector Laboratories, S-1000) and 0.1% Triton X-100 (Sigma-Aldrich, T9284) in PBS for 60 minutes at room temperature, followed by incubation with the primary antibodies chicken anti-GFP (1:1,000; Abcam, ab13970) for detecting GFP/YFP and GCaMP6f/GCaMP7f/GCaMP8f and rabbit anti-RFP (1:1,000; Abcam, ab62341) and rabbit anti-mCherry (1:1,000; Abcam, ab183628) for detecting tdTomato/mCherry at 4 °C for overnight. After washing out excessive primary antibodies three times with PBS, the sections were incubated with the secondary antibodies (1:500; Alexa Fluor 488-conjugated goat anti-chicken, Invitrogen, A-11039, and Alexa Fluor 568-conjugated goat anti-Rb, Invitrogen, A-11011) for 2 hours at room temperature. After three washes with PBS, the sections were mounted on slides and coverslipped with HardSet Antifade Mounting Medium with DAPI (H-1500, VECTASHIELD). Slices were imaged on a Zeiss Axio Observer Z1 fluorescence microscope with ×10 objective. The images were acquired and processed using ZEN 2012 Blue software (Zeiss). For 3,3’-diaminobenzidine (DAB) staining, sections were blocked with 10% normal goat serum (Vector Laboratories, S-1000) and 0.1% Triton X-100 (Sigma-Aldrich, T9284) for 1 hour at room temperature, followed by incubation with a primary antibody against TH (1:4,000; Millipore/Chemicon, ab152) overnight at 4 °C. After washing out excessive primary antibodies three times with PBS, sections were then incubated with a biotinylated secondary antibody (1:200; Vector Laboratories, BA-1000) for 1 hour, followed by incubation in avidin-biotin peroxidase complex (Vector Laboratories, PK6100) for 1 hour, and immunoreactivity was detected with DAB (Sigma-Aldrich, D5905) for 40−80 seconds. After thorough washing with PBS, the sections were mounted on slides and then dehydrated and cleared with ethanol and xylenes prior to coverslipping. Tissue was imaged at the NIEHS Image Analysis Group using an Aperio AT2 slide scanner (Leica Biosystems).

### TH^+^ cell counting and quantification of TH^+^ terminal density

RGB color images were analyzed using ImageJ (1.8.0_60) or FIJI (ImageJ 1.52e) for TH^+^ cell counting in the midbrain and for quantification of dopamine axon terminal density in the dorsolateral striatum with the experimenter blinded to the genotypes or treatment conditions. For TH^+^ cell counting, after the image was split into its RGB components, the green channel was used for analysis because it gave the highest contrast in the DAB staining. The image for the green channel was inverted; then, an eliminate maxima filter (size 7) from the Fast Filters plugin, a Gaussian blur filter (size 7) and background subtraction (size 60) were used to identify the DAB-stained cell bodies. Next, a mask was created by using the default automatic threshold where we performed a watershed algorithm for segmentation and counted each particle over 100 pixels with the Analyze Particles feature. Results of the counted features were then confirmed visually by overlaying the region of interest for each counted object back to the original DAB-stained image to ensure accuracy. For quantification of TH^+^ terminal density, the image was first split into its RGB components from which an intensity image for DAB was extracted, converted to grayscale and inverted. The dorsolateral striatum was outlined, and the threshold values for pixel intensity were set from 45 to 255. To calculate the optical density, gray pixel values of images were calibrated to a density standard from an optical density grayscale.

### Statistics and reproducibility

One-way ANOVA, two-way ANOVA followed by multiple comparisons and two-tailed unpaired and paired *t*-tests were carried out using GraphPad Prism 8 (GraphPad Software). Results of the statistical analyses, including mean ± s.e.m., sample sizes and *P* values, are indicated either in the text or the figure legends. Age-matched and sex-matched mice were randomly assigned to groups for all experiments. No statistical methods were used to predetermine sample sizes. Instead, group sizes were selected based on similar studies. Data collection was conducted in a blinded manner. No data were excluded from the analyses. Several 6-OHDA-lesioned mice and one MitoPark mouse were removed from the study after reaching humane endpoints, as defined in our Animal Study Protocol (ASP 2021-0014), prior to study completion. All 6-OHDA-lesioned mice underwent immunohistochemical staining to verify dopaminergic neuron loss. All mice that received viral injections were assessed for viral expression and fiber tract placement. For the representative immunofluorescence and immunohistochemical images shown in the figures, the staining experiments were performed on at least three mice from each group.

### Reporting summary

Further information on research design is available in the Nature Portfolio Reporting Summary linked to this article.

## Online content

Any methods, additional references, Nature Portfolio reporting summaries, source data, extended data, supplementary information, acknowledgements, peer review information; details of author contributions and competing interests; and statements of data and code availability are available at 10.1038/s41593-025-02088-w.

## Supplementary information

Supplementary Information Supplementary Figs. 1 and 2.

Reporting Summary

Supplementary Video 1 A 15-minute recording showing spontaneous rotational behavior in a unilaterally (left side) 6-OHDA-lesioned Vglut2-Cre mouse before, during, and after therapeutic DBS (150 µA) applied to the ipsilateral STN. The video is played at 10X normal speed. Pre-DBS (0–5 min): The mouse exhibited 24 leftward rotations and 0 rightward rotations. DBS ON (5–10 min): The mouse exhibited 6 leftward rotations and 10 rightward rotations, indicating a reduction in pathological turning bias. Post-DBS (10–15 min): The mouse reverted to 23 leftward rotations and 0 rightward rotations. This video illustrates the reversible therapeutic effect of STN DBS on pathological rotational behavior in 6-OHDA-lesioned PD mice. (Related to Fig. 4)

Supplementary Video 2 The same video as Supplementary Video 1 but played at normal speed. (Related to Fig. 4)

Supplementary Video 3 Two 5-minute recordings showing spontaneous rotational behavior in a unilaterally (right side) 6-OHDA-lesioned Vglut2-Cre mouse with Gi DREADD expression in the ipsilateral STN, following vehicle or CNO treatment. The video is played at 5X normal speed. The mouse exhibited 12 rightward rotations and 0 leftward rotations after vehicle treatment, and 4 rightward rotations and 4 leftward rotations after CNO during the 5-min recording sessions, indicating a reduction in pathological turning bias by chemogenetic inhibition of STN (chemogenetic DBS) in 6-OHDA-lesioned PD mice. (Related to Fig. 5)

Supplementary Video 4 Two 5-minute recordings showing spontaneous rotational behavior in a unilaterally (left side) 6-OHDA-lesioned Vglut2-Cre mouse with Gq DREADD expression in the ipsilateral STN, following vehicle or CNO treatment. The video is played at 5X normal speed. The mouse exhibited 14 leftward rotations and 0 rightward rotations after vehicle treatment, and 38 leftward rotations and 0 rightward rotations after CNO during the 5-min recording sessions, indicating that chemogenetic excitation of STN significantly exacerbates the pathological turning bias in 6-OHDA-lesioned PD mice. (Related to Fig. 5)

Supplementary Video 5 Four 5-minute recordings to show the locomotor activity of littermate control and MitoPark mice with bilateral expression of Gi DREADD in the STN before and after CNO treatment. The video is played at 10X normal speed. (Related to Fig. 6)

Supplementary Video 6 Comparison between chemogenetic DBS and electrical DBS in the same MitoPark PD mouse. The four windows show 5-minute recordings of a MitoPark mouse with bilateral expression of Gi DREADD in the STN and bilateral implantation of electrode-optical fiber hybrid probes in the STN at baseline (top left), 30 min after an i.p. injection of vehicle (top right) or 3 mg/kg CNO (bottom left), and during electrical DBS (bottom right, current intensity: 150 µA). The video is played at 10X normal speed. (Related to Fig. 7)

Supplementary Data 1 Statistical source data.

Supplementary Data 2 Statistical source data.

## Source data

Source Data Fig. 1 Statistical source data.

Source Data Fig. 2 Statistical source data.

Source Data Fig. 3 Statistical source data.

Source Data Fig. 4 Statistical source data.

Source Data Fig. 5 Statistical source data.

Source Data Fig. 6 Statistical source data.

Source Data Fig. 7 Statistical source data.

Source Data Extended Data Fig. 2 Statistical source data.

Source Data Extended Data Fig. 3 Statistical source data.

Source Data Extended Data Fig. 4 Statistical source sata.

Source Data Extended Data Fig. 5 Statistical source data.

Source Data Extended Data Fig. 6 Statistical source data.

Source Data Extended Data Fig. 7 Statistical source data.

Source Data Extended Data Fig. 8 Statistical source data.

Source Data Extended Data Fig. 9 Statistical source data.

Source Data Extended Data Fig. 10 Statistical source data.

## Data Availability

All data are available from the corresponding authors upon reasonable request. Source data are provided with this paper.

## Code availability

The linear spectral unmixing algorithm used in this study can be downloaded at https://www.niehs.nih.gov/research/atniehs/labs/ln/pi/iv/tools.

## References

[1] Lozano, A. M. et al. Deep brain stimulation: current challenges and future directions. *Nat. Rev. Neurol.* **15**, 148–160 (2019). 30683913 10.1038/s41582-018-0128-2 PMC6397644

[2] Limousin, P. et al. Effect of parkinsonian signs and symptoms of bilateral subthalamic nucleus stimulation. *Lancet* **345**, 91–95 (1995). 7815888 10.1016/s0140-6736(95)90062-4

[3] Follett, K. A. et al. Pallidal versus subthalamic deep-brain stimulation for Parkinson’s disease. *N. Engl. J. Med.* **362**, 2077–2091 (2010). 20519680 10.1056/NEJMoa0907083

[4] Odekerken, V. J. et al. Subthalamic nucleus versus globus pallidus bilateral deep brain stimulation for advanced Parkinson’s disease (NSTAPS study): a randomised controlled trial. *Lancet Neurol* **12**, 37–44 (2013). 23168021 10.1016/S1474-4422(12)70264-8

[5] Herrington, T. M., Cheng, J. J. & Eskandar, E. N. Mechanisms of deep brain stimulation. *J. Neurophysiol.* **115**, 19–38 (2016). 26510756 10.1152/jn.00281.2015 PMC4760496

[6] Ashkan, K., Rogers, P., Bergman, H. & Ughratdar, I. Insights into the mechanisms of deep brain stimulation. *Nat. Rev. Neurol.* **13**, 548–554 (2017). 28752857 10.1038/nrneurol.2017.105

[7] Neumann, W. J., Steiner, L. A. & Milosevic, L. Neurophysiological mechanisms of deep brain stimulation across spatiotemporal resolutions. *Brain* **146**, 4456–4468 (2023). 37450573 10.1093/brain/awad239 PMC10629774

[8] Chiken, S. & Nambu, A. Mechanism of deep brain stimulation: inhibition, excitation, or disruption? *Neuroscientist* **22**, 313–322 (2016). 25888630 10.1177/1073858415581986 PMC4871171

[9] Benabid, A. L. et al. Acute and long-term effects of subthalamic nucleus stimulation in Parkinson’s disease. *Stereotact. Funct. Neurosurg.* **62**, 76–84 (1994). 7631092 10.1159/000098600

[10] Bergman, H., Wichmann, T., Karmon, B. & DeLong, M. R. The primate subthalamic nucleus. II. Neuronal activity in the MPTP model of parkinsonism. *J. Neurophysiol.* **72**, 507–520 (1994). 7983515 10.1152/jn.1994.72.2.507

[11] Bergman, H., Wichmann, T. & DeLong, M. R. Reversal of experimental parkinsonism by lesions of the subthalamic nucleus. *Science* **249**, 1436–1438 (1990). 2402638 10.1126/science.2402638

[12] Steigerwald, F. et al. Neuronal activity of the human subthalamic nucleus in the parkinsonian and nonparkinsonian state. *J. Neurophysiol.* **100**, 2515–2524 (2008). 18701754 10.1152/jn.90574.2008

[13] Benazzouz, A. et al. Effect of high-frequency stimulation of the subthalamic nucleus on the neuronal activities of the substantia nigra pars reticulata and ventrolateral nucleus of the thalamus in the rat. *Neuroscience* **99**, 289–295 (2000). 10938434 10.1016/s0306-4522(00)00199-8

[14] Benazzouz, A., Piallat, B., Pollak, P. & Benabid, A. L. Responses of substantia nigra pars reticulata and globus pallidus complex to high frequency stimulation of the subthalamic nucleus in rats: electrophysiological data. *Neurosci. Lett.* **189**, 77–80 (1995). 7609923 10.1016/0304-3940(95)11455-6

[15] Hashimoto, T., Elder, C. M., Okun, M. S., Patrick, S. K. & Vitek, J. L. Stimulation of the subthalamic nucleus changes the firing pattern of pallidal neurons. *J. Neurosci.* **23**, 1916–1923 (2003). 12629196 10.1523/JNEUROSCI.23-05-01916.2003 PMC6741976

[16] Maurice, N., Thierry, A. M., Glowinski, J. & Deniau, J. M. Spontaneous and evoked activity of substantia nigra pars reticulata neurons during high-frequency stimulation of the subthalamic nucleus. *J. Neurosci.* **23**, 9929–9936 (2003). 14586023 10.1523/JNEUROSCI.23-30-09929.2003 PMC6740874

[17] Johnson, L. A. et al. Direct activation of primary motor cortex during subthalamic but not pallidal deep brain stimulation. *J. Neurosci.* **40**, 2166–2177 (2020). 32019827 10.1523/JNEUROSCI.2480-19.2020 PMC7055133

[18] Gradinaru, V., Mogri, M., Thompson, K. R., Henderson, J. M. & Deisseroth, K. Optical deconstruction of parkinsonian neural circuitry. *Science* **324**, 354–359 (2009). 19299587 10.1126/science.1167093 PMC6744370

[19] Yoon, H. H. et al. Optogenetic inactivation of the subthalamic nucleus improves forelimb akinesia in a rat model of Parkinson disease. *Neurosurgery* **74**, 533–540 (2014). 24463495 10.1227/NEU.0000000000000297

[20] McIver, E. L. et al. Maladaptive downregulation of autonomous subthalamic nucleus activity following the loss of midbrain dopamine neurons. *Cell Rep.* **28**, 992–1002 (2019). 31340159 10.1016/j.celrep.2019.06.076 PMC6699776

[21] Xie, C., Power, J. & Prasad, A. A. Bidirectional optogenetic modulation of the subthalamic nucleus in a rodent model of Parkinson’s disease. *Front. Neurosci.* **16**, 848821 (2022). 35655750 10.3389/fnins.2022.848821 PMC9152094

[22] Meng, C. et al. Spectrally resolved fiber photometry for multi-component analysis of brain circuits. *Neuron* **98**, 707–717 (2018). 29731250 10.1016/j.neuron.2018.04.012 PMC5957785

[23] Soh, D., Ten Brinke, T. R., Lozano, A. M. & Fasano, A. Therapeutic window of deep brain stimulation using cathodic monopolar, bipolar, semi-bipolar, and anodic stimulation. *Neuromodulation* **22**, 451–455 (2019). 30951239 10.1111/ner.12957

[24] Knorr, S. et al. Experimental deep brain stimulation in rodent models of movement disorders. *Exp. Neurol.* **348**, 113926 (2022). 34793784 10.1016/j.expneurol.2021.113926

[25] McIntyre, C. C., Grill, W. M., Sherman, D. L. & Thakor, N. V. Cellular effects of deep brain stimulation: model-based analysis of activation and inhibition. *J. Neurophysiol.* **91**, 1457–1469 (2004). 14668299 10.1152/jn.00989.2003

[26] Alexander, G. E. & Crutcher, M. D. Functional architecture of basal ganglia circuits: neural substrates of parallel processing. *Trends Neurosci.* **13**, 266–271 (1990). 1695401 10.1016/0166-2236(90)90107-l

[27] Makani, S. & Chesler, M. Rapid rise of extracellular pH evoked by neural activity is generated by the plasma membrane calcium ATPase. *J. Neurophysiol.* **103**, 667–676 (2010). 19939954 10.1152/jn.00948.2009 PMC2822688

[28] Zhang, W. T. et al. Spectral fiber photometry derives hemoglobin concentration changes for accurate measurement of fluorescent sensor activity. Cell Rep. *Methods* **2**, 100243 (2022). 10.1016/j.crmeth.2022.100243 PMC9308135 35880016

[29] Moro, E. et al. The impact on Parkinson’s disease of electrical parameter settings in STN stimulation. *Neurology* **59**, 706–713 (2002). 12221161 10.1212/wnl.59.5.706

[30] Eusebio, A. et al. Effects of low-frequency stimulation of the subthalamic nucleus on movement in Parkinson’s disease. *Exp. Neurol.* **209**, 125–130 (2008). 17950279 10.1016/j.expneurol.2007.09.007 PMC2288636

[31] Benabid, A. L. Deep brain stimulation for Parkinson’s disease. *Curr. Opin. Neurobiol.* **13**, 696–706 (2003). 14662371 10.1016/j.conb.2003.11.001

[32] Ekstrand, M. I. et al. Progressive parkinsonism in mice with respiratory-chain-deficient dopamine neurons. *Proc. Natl Acad. Sci. USA* **104**, 1325–1330 (2007). 17227870 10.1073/pnas.0605208103 PMC1783140

[33] Alabi, A. A. & Tsien, R. W. Synaptic vesicle pools and dynamics. *Cold Spring Harb. Perspect. Biol.* **4**, a013680 (2012). 22745285 10.1101/cshperspect.a013680 PMC3405865

[34] Steiner, L. A. et al. Connectivity and dynamics underlying synaptic control of the subthalamic nucleus. *J. Neurosci.* **39**, 2470–2481 (2019). 30700533 10.1523/JNEUROSCI.1642-18.2019 PMC6435833

[35] Galarreta, M. & Hestrin, S. Frequency-dependent synaptic depression and the balance of excitation and inhibition in the neocortex. *Nat. Neurosci.* **1**, 587–594 (1998). 10196566 10.1038/2822

[36] Varela, J. A., Song, S., Turrigiano, G. G. & Nelson, S. B. Differential depression at excitatory and inhibitory synapses in visual cortex. *J. Neurosci.* **19**, 4293–4304 (1999). 10341233 10.1523/JNEUROSCI.19-11-04293.1999 PMC6782599

[37] Chen, C., Arai, I., Satterfield, R., Young, S. M. Jr. & Jonas, P. Synaptotagmin 2 is the fast Ca^2+^ sensor at a central inhibitory synapse. *Cell Rep.* **18**, 723–736 (2017). 28099850 10.1016/j.celrep.2016.12.067 PMC5276807

[38] Kraushaar, U. & Jonas, P. Efficacy and stability of quantal GABA release at a hippocampal interneuron-principal neuron synapse. *J. Neurosci.* **20**, 5594–5607 (2000). 10908596 10.1523/JNEUROSCI.20-15-05594.2000 PMC6772523

[39] Zucker, R. S. & Regehr, W. G. Short-term synaptic plasticity. *Annu. Rev. Physiol.* **64**, 355–405 (2002). 11826273 10.1146/annurev.physiol.64.092501.114547

[40] Temperli, P. et al. How do parkinsonian signs return after discontinuation of subthalamic DBS? *Neurology* **60**, 78–81 (2003). 12525722 10.1212/wnl.60.1.78

[41] Lopiano, L. et al. Temporal changes in movement time during the switch of the stimulators in Parkinson’s disease patients treated by subthalamic nucleus stimulation. *Eur. Neurol.* **50**, 94–99 (2003). 12944714 10.1159/000072506

[42] Kuhn, A. A. et al. High-frequency stimulation of the subthalamic nucleus suppresses oscillatory beta activity in patients with Parkinson’s disease in parallel with improvement in motor performance. *J. Neurosci.* **28**, 6165–6173 (2008). 18550758 10.1523/JNEUROSCI.0282-08.2008 PMC6670522

[43] Erez, Y., Tischler, H., Moran, A. & Bar-Gad, I. Generalized framework for stimulus artifact removal. *J. Neurosci. Methods* **191**, 45–59 (2010). 20542059 10.1016/j.jneumeth.2010.06.005

[44] Carlson, J. D., Cleary, D. R., Cetas, J. S., Heinricher, M. M. & Burchiel, K. J. Deep brain stimulation does not silence neurons in subthalamic nucleus in Parkinson’s patients. *J. Neurophysiol.* **103**, 962–967 (2010). 19955287 10.1152/jn.00363.2009 PMC3141810

[45] Schor, J. S. et al. Therapeutic deep brain stimulation disrupts movement-related subthalamic nucleus activity in parkinsonian mice. *eLife* **11**, e75253 (2022). 35786442 10.7554/eLife.75253 PMC9342952

[46] McIntyre, C. C. & Grill, W. M. Selective microstimulation of central nervous system neurons. *Ann. Biomed. Eng.* **28**, 219–233 (2000). 10784087 10.1114/1.262

[47] Chaturvedi, A., Lujan, J. L. & McIntyre, C. C. Artificial neural network based characterization of the volume of tissue activated during deep brain stimulation. *J. Neural Eng.* **10**, 056023 (2013). 24060691 10.1088/1741-2560/10/5/056023 PMC4115460

[48] Chakraborty, D., Truong, D. Q., Bikson, M. & Kaphzan, H. Neuromodulation of axon terminals. *Cereb. Cortex* **28**, 2786–2794 (2018). 28655149 10.1093/cercor/bhx158 PMC6041977

[49] Windels, F. et al. Effects of high frequency stimulation of subthalamic nucleus on extracellular glutamate and GABA in substantia nigra and globus pallidus in the normal rat. *Eur. J. Neurosci.* **12**, 4141–4146 (2000). 11069610 10.1046/j.1460-9568.2000.00296.x

[50] Francardo, V. et al. Impact of the lesion procedure on the profiles of motor impairment and molecular responsiveness to L-DOPA in the 6-hydroxydopamine mouse model of Parkinson’s disease. *Neurobiol. Dis.* **42**, 327–340 (2011). 21310234 10.1016/j.nbd.2011.01.024

[51] Levy, R. et al. Lidocaine and muscimol microinjections in subthalamic nucleus reverse Parkinsonian symptoms. *Brain* **124**, 2105–2118 (2001). 11571226 10.1093/brain/124.10.2105

[52] Petri, D., Pum, M., Vesper, J., Huston, J. P. & Schnitzler, A. GABAA-receptor activation in the subthalamic nucleus compensates behavioral asymmetries in the hemiparkinsonian rat. *Behav. Brain Res.* **252**, 58–67 (2013). 23727148 10.1016/j.bbr.2013.05.044

[53] Patel, N. K. et al. Unilateral subthalamotomy in the treatment of Parkinson’s disease. *Brain* **126**, 1136–1145 (2003). 12690053 10.1093/brain/awg111

[54] Roth, B. L. DREADDs for neuroscientists. *Neuron* **89**, 683–694 (2016). 26889809 10.1016/j.neuron.2016.01.040 PMC4759656

[55] Assaf, F. & Schiller, Y. A chemogenetic approach for treating experimental Parkinson’s disease. *Mov. Disord.* **34**, 469–479 (2019). 30536778 10.1002/mds.27554

[56] Chen, Y. et al. Circuit-specific gene therapy reverses core symptoms in a primate Parkinson’s disease model. *Cell* **186**, 5394–5410 (2023). 37922901 10.1016/j.cell.2023.10.004

[57] Gompf, H. S., Budygin, E. A., Fuller, P. M. & Bass, C. E. Targeted genetic manipulations of neuronal subtypes using promoter-specific combinatorial AAVs in wild-type animals. *Front. Behav. Neurosci.* **9**, 152 (2015). 26190981 10.3389/fnbeh.2015.00152 PMC4488755

[58] Qian, Y. et al. Programmable RNA sensing for cell monitoring and manipulation. *Nature* **610**, 713–721 (2022). 36198803 10.1038/s41586-022-05280-1 PMC10348343

[59] Krauss, J. K. et al. Technology of deep brain stimulation: current status and future directions. *Nat. Rev. Neurol.* **17**, 75–87 (2021). 33244188 10.1038/s41582-020-00426-z PMC7116699

[60] Spix, T. A. et al. Population-specific neuromodulation prolongs therapeutic benefits of deep brain stimulation. *Science* **374**, 201–206 (2021). 34618556 10.1126/science.abi7852 PMC11098594

[61] Zhou, J., Li, J., Papaneri, A. B., Kobzar, N. P. & Cui, G. Dopamine Neuron Challenge Test for early detection of Parkinson’s disease. *npj Parkinsons Dis.* **7**, 116 (2021). 34916526 10.1038/s41531-021-00261-z PMC8677804

[62] Cui, G. et al. Deep brain optical measurements of cell type-specific neural activity in behaving mice. *Nat. Protoc.* **9**, 1213–1228 (2014). 24784819 10.1038/nprot.2014.080 PMC4100551

[63] Chen, T. W. et al. Ultrasensitive fluorescent proteins for imaging neuronal activity. *Nature* **499**, 295–300 (2013). 23868258 10.1038/nature12354 PMC3777791

[64] Dana, H. et al. High-performance calcium sensors for imaging activity in neuronal populations and microcompartments. *Nat. Methods* **16**, 649–657 (2019). 31209382 10.1038/s41592-019-0435-6

[65] Marvin, J. S. et al. Stability, affinity, and chromatic variants of the glutamate sensor iGluSnFR. *Nat. Methods* **15**, 936–939 (2018). 30377363 10.1038/s41592-018-0171-3 PMC6394230

[66] Marvin, J. S. et al. A genetically encoded fluorescent sensor for in vivo imaging of GABA. *Nat. Methods* **16**, 763–770 (2019). 31308547 10.1038/s41592-019-0471-2

[67] Zhang, Y. et al. Fast and sensitive GCaMP calcium indicators for imaging neural populations. *Nature* **615**, 884–891 (2023). 36922596 10.1038/s41586-023-05828-9 PMC10060165

