# Supplementary material for: Differential synaptic depression mediates the therapeutic effect of deep brain stimulation
Source: Nat Neurosci. 2025 Oct 16;28(12):2575–87. doi: 10.1038/s41593-025-02088-w (PMC12672378; doi:10.1038/s41593-025-02088-w)
Supplement: Supplementary file 1 — Supplementary Figs. 1 and 2. [file 41593_2025_2088_MOESM1_ESM.pdf]

nature neuroscience

Supplementary information https://doi.org/10.1038/s41593-025-02088-w

Differential synaptic depression mediates the therapeutic effect of deep brain stimulation

In the format provided by the authors and unedited

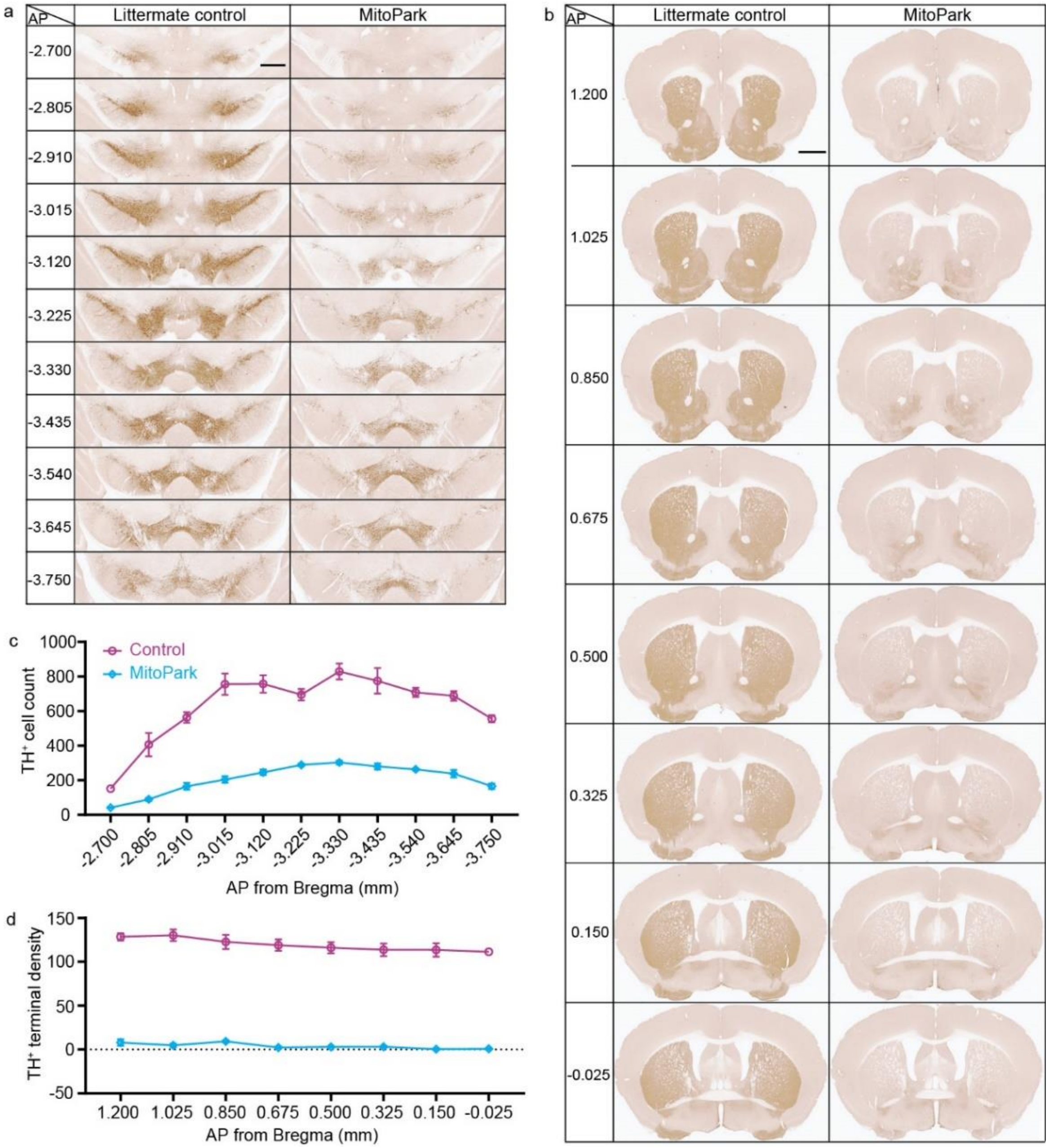

Supplementary Figure 1. TH staining and quantification to validate the degeneration of DA neurons in 29-week-old MitoPark mice.

a, b, Representative images of DAB staining of TH+ cells in the ventral midbrain (a) and TH+ terminals density in the striatum (b). Scale bars: 500 µm for midbrain and 1mm for the striatum. c, Summary of the TH^+ cell counting in the ventral midbrain from anterior to posterior sections of littermate control and MitoPark mice. d, Summary of the TH^+ cell terminal density in the striatum from anterior to posterior sections of littermate control and MitoPark mice. For c and d, all data are plotted as mean ± SEM. n = 5 mice for littermate control group, n = 7 mice for MitoPark mice group, including both males and females.

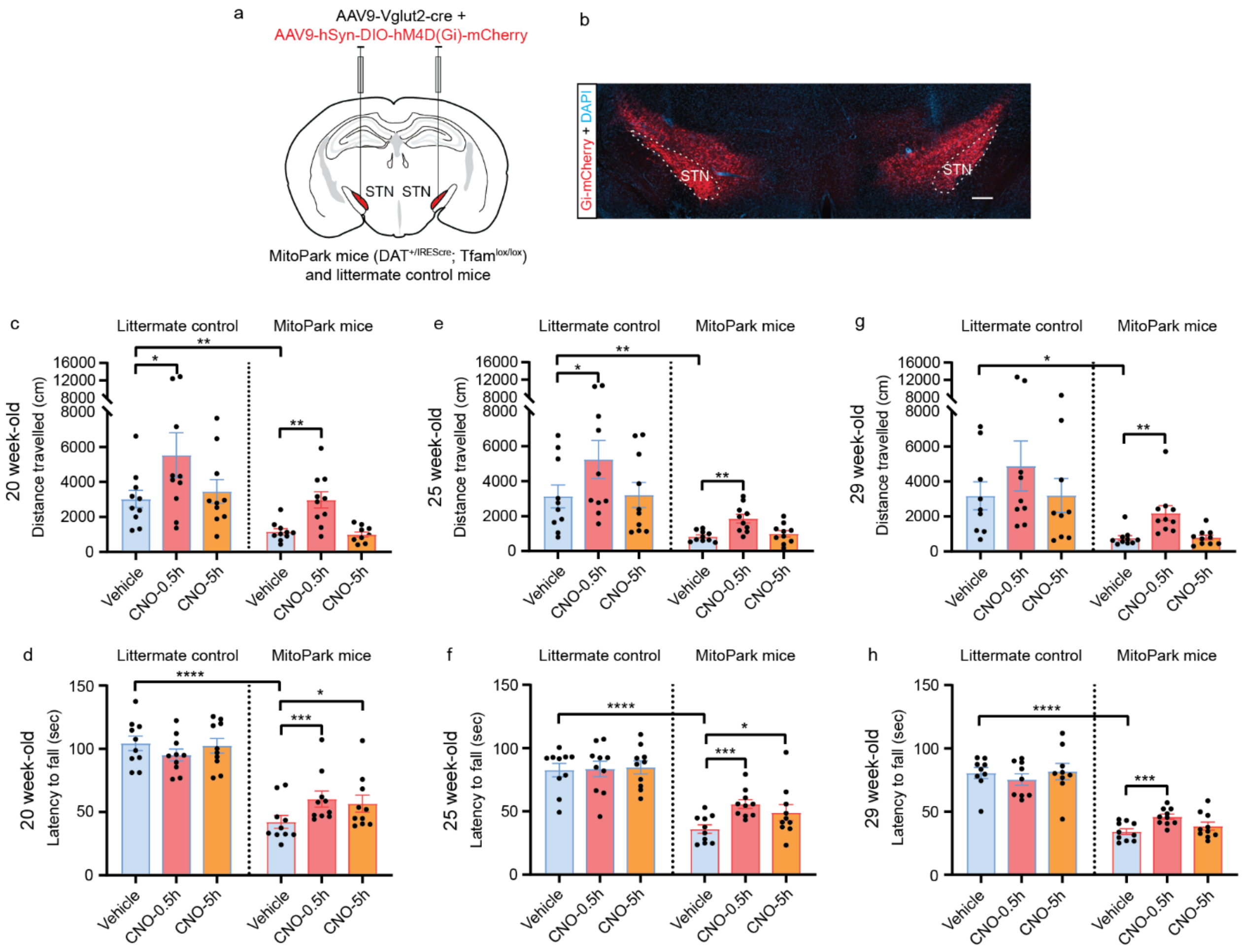

Supplementary Figure 2. Co-injection of AAV9-Vglut2-cre and AAV9-hSyn-DIO-hM4D(Gi)-mCherry did not achieve specific expression of hM4D(Gi)-mCherry in STN neurons in MitoPark mice.

a, Schematic illustration to show bilateral viral expression of hM4D(Gi)-mCherry by microinjection of viral mixtures containing AAV9-Vglut2-cre and AAV9-hSyn-DIO-hM4D(Gi)-mCherry in the STN MitoPark mice and littermate control mice. b, Immunofluorescence staining to show the expression of hM4D(Gi)-mCherry in the STN and nearby regions. Scale bar: 200 µm. c, e, g, Open field tests to show the total traveled distance in control and MitoPark mice expressing hM4D(Gi)-mCherry in the STN and nearby regions, measured longitudinally at the age of 20 weeks (c), 25 weeks (e) and 29 weeks (g). d, f, h, Rotarod tests to show the latency to fall in control and MitoPark mice expressing hM4D(Gi)-mCherry in the STN and nearby regions, measured longitudinally at the age of 20 weeks (d), 25 weeks (f) and 29 weeks (h). * $p < 0.05$, ** $p < 0.01$, *** $p < 0.001$, **** $p < 0.0001$ (For c: Littermate-Vehicle vs. MitoPark-Vehicle, $p = 0.0047$; Littermate-Vehicle vs. Littermate-CNO-0.5h, $p = 0.0466$; MitoPark- Vehicle vs. MitoPark-CNO-0.5h, $p = 0.0055$; for d: Littermate-Vehicle vs. MitoPark-Vehicle, $p < 0.0001$; MitoPark-Vehicle vs. MitoPark-CNO-0.5h, $p = 0.0008$; MitoPark-Vehicle vs. MitoPark-CNO-5h, $p = 0.0148$; for e: Littermate-Vehicle vs. MitoPark-Vehicle, $p = 0.0069$; Littermate-Vehicle vs. Littermate-CNO-0.5h, $p = 0.0206$; MitoPark-Vehicle vs. MitoPark-CNO-0.5h, $p = 0.0030$; for f:

Littermate-Vehicle vs. MitoPark-Vehicle, $p < 0.0001$; MitoPark-Vehicle vs. MitoPark-CNO-0.5h, $p = 0.0003$; MitoPark-Vehicle vs. MitoPark-CNO-5h, $p = 0.0458$; for g: Littermate-Vehicle vs. MitoPark-Vehicle, $p = 0.0164$; MitoPark-Vehicle vs. MitoPark-CNO-0.5h, $p = 0.0025$; for h: Littermate-Vehicle vs. MitoPark-Vehicle, $p < 0.0001$; MitoPark-Vehicle vs. MitoPark-CNO-0.5h, $p = 0.0004$). Two-way ANOVA followed by Tukey's multiple comparisons test. All data are plotted as mean ± SEM. n = 9 mice for 29-week-old littermate control group, n = 10 mice for all other groups, including both males and females.
